# Repurposing the antipsychotic drug amisulpride for targeting synovial fibroblast activation in arthritis

**DOI:** 10.1172/jci.insight.165024

**Published:** 2023-05-08

**Authors:** Dimitra Papadopoulou, Fani Roumelioti, Christos Tzaferis, Panagiotis Chouvardas, Anna-Kathrine Pedersen, Filippos Charalampous, Eleni Christodoulou-Vafeiadou, Lydia Ntari, Niki Karagianni, Maria C. Denis, Jesper V. Olsen, Alexios N. Matralis, George Kollias

**Affiliations:** 1 Institute for Bioinnovation, Biomedical Sciences Research Centre Alexander Fleming”, Vari, Greece.; 2 Department of Physiology, School of Medicine, National and Kapodistrian University of Athens, Athens, Greece.; 3 Novo Nordisk Foundation Center for Protein Research, Faculty of Health and Medical Sciences, University of Copenhagen, Copenhagen, Denmark.; 4 Biomedcode Hellas SA, Vari, Greece.; 5 Center of New Biotechnologies & Precision Medicine, School of Medicine, National and Kapodistrian University of Athens, Athens, Greece.

**Keywords:** Therapeutics, Arthritis, Mouse models

## Abstract

Synovial fibroblasts (SFs) are key pathogenic drivers in rheumatoid arthritis (RA). Their in vivo activation by TNF is sufficient to orchestrate full arthritic pathogenesis in animal models, and TNF blockade proved efficacious for a high percentage of patients with RA albeit coinducing rare but serious side effects. Aiming to find new potent therapeutics, we applied the L1000CDS^2^ search engine, to repurpose drugs that could reverse the pathogenic expression signature of arthritogenic human TNF–transgenic (*hTNFtg*) SFs. We identified a neuroleptic drug, namely amisulpride, which reduced SFs’ inflammatory potential while decreasing the clinical score of *hTNFtg* polyarthritis. Notably, we found that amisulpride function was neither through its known targets dopamine receptors D2 and D3 and serotonin receptor 7 nor through TNF–TNF receptor I binding inhibition. Through a click chemistry approach, potentially novel targets of amisulpride were identified, which were further validated to repress *hTNFtg* SFs’ inflammatory potential ex vivo (*Ascc3* and *Sec62*), while phosphoproteomics analysis revealed that treatment altered important fibroblast activation pathways, such as adhesion. Thus, amisulpride could prove beneficial to patients experiencing RA and the often-accompanying comorbid dysthymia, reducing SF pathogenicity along with its antidepressive activity, serving further as a “lead” compound for the development of novel therapeutics against fibroblast activation.

## Graphical abstract

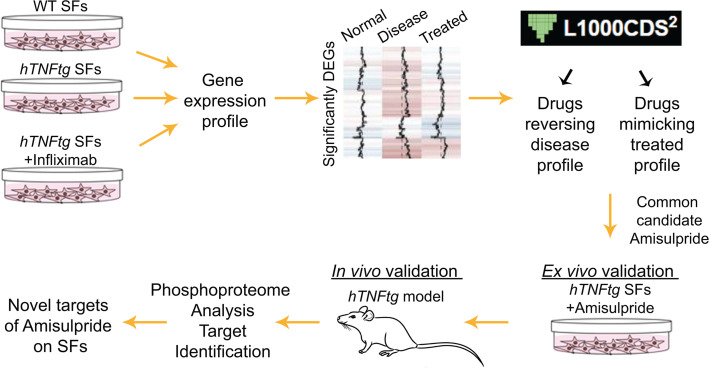

## Introduction

Rheumatoid arthritis (RA) is a chronic inflammatory disease characterized by swelling and gradual destruction of the joints. Synovial fibroblasts (SFs) are one of the main cell types that hyperproliferate during RA progression, producing inflammatory cytokines/chemokines and degrading metalloproteinases, leading progressively to increased joint inflammation, stiffness, and pain (1). We have shown that mice overexpressing TNF, by carrying a human TNF transgene (*hTNFtg* mice) (2) or by deletion of ARE elements in the endogenous *Tnf* gene (*TNF*^ΔARE^ mice) (3), spontaneously develop chronic polyarthritis, with histological manifestations fully resembling human RA. Notably, arthritic pathology in both models develops independently of the adaptive immune response highlighting the dominant role of the innate/stromal compartment in the development of disease (3, 4). Most importantly, TNF signaling via TNF receptor I (TNFR1) in SFs was found to be both required and necessary for the orchestration of full RA-like pathology (5, 6). Interestingly, *hTNFtg* SFs have been found to highly correlate with RA human fibroblast-like synoviocytes (FLS) both at the bulk (7) and at the single-cell RNA levels (8–10). Together, these findings established in principle the dominant in vivo role of SFs in the initiation and progression of chronic polyarthritis and suggested a mechanism that may also explain, at least in part, the development of joint pathology in the human disease.

Current first-line therapies against RA, including disease-modifying antirheumatic drugs (DMARDs), such as methotrexate, and targeted synthetic DMARDs inhibiting several kinases, such as Janus kinases or mitogen-activated protein kinase (MAPK), offer significant clinical benefits (11). However, although these therapeutics reduce the disease symptoms, patients progressively may lose response. In more severe cases, anti–TNF/IL-6 biologics are prescribed, but their higher therapeutic potential is counterbalanced by decreased patient compliance due to invasive administration, as well as by a plethora of side effects, such as the gradual development of immunodeficiencies or autoimmune flares and the production of antidrug antibodies, leading to consequent lower responses to treatment (12).

Herein, aiming to identify new potential therapeutics for SF deactivation in RA, we searched for compounds in a repurposing setting, as this could provide new candidates already assessed for toxicity, formulation, and route of administration (13). To this end, we used the L1000CDS^2^ search engine (14), which has been employed successfully in the past for repurposing small molecules as therapeutics for several diseases (15), to identify compounds that could potentially reverse the pathogenic gene expression signature of *hTNFtg* SFs, while mimicking the ex vivo effect of a known anti-TNF biologic, infliximab. We have identified a commercially available neuroleptic medicine, namely amisulpride, that was validated here to downregulate *hTNFtg* SFs’ activation by reducing both their inflammatory and adhesive potential, while ameliorating clinical *hTNFtg* pathology. Interestingly, the mechanism through which amisulpride exerts its activity was found to be independent of the main reported targets of the drug, dopamine receptor D2 and D3 (DRD2 and DRD3) and serotonin receptor 7 (HTR7). Notably, 5 potential targets of amisulpride on fibroblasts were identified, 2 of which, namely *Ascc3* and *Sec62*, were further validated to repress *hTNFtg* SFs’ inflammatory potential. Thus, amisulpride could provide benefit to patients experiencing RA and comorbid dysthymia, reducing SFs’ pathogenicity in parallel with its antidepressive activity. Amisulpride may also be used as a “lead” compound for the development of novel compounds targeting fibroblast activation in relevant diseases, beyond RA.

## Results

### Identification of amisulpride as a modifier of hTNFtg SF activation.

To identify new drug candidates as potential therapeutics for RA, the publicly available search engine L1000CDS^2^, which explores a set of different combinations of treatments and cell lines, to find perturbations that either mimic or reverse the user’s input signature, was used (14). Initially 3′ mRNA sequencing of SFs isolated from 8-week-old *hTNFtg* mice (established disease) and wild-type (WT) controls was performed, and the differentially expressed genes were identified (Supplemental Figures 1A and 2B and Supplemental Data 1; supplemental material available online with this article; https://doi.org/10.1172/jci.insight.165024DS1). The results of this analysis were then combined with data already published (7), aiming at precisely determining those genes that are commonly deregulated at all 3 stages of the disease (early, established, late) when compared with the WT control. Additionally, the expression profile of 8-week *hTNFtg* SFs after a 48-hour treatment with 1 μg/mL infliximab, a widely used anti-TNF biologic, was compared with the relevant untreated *hTNFtg* SFs (Supplemental Figure 1B, Supplemental Figure 2B, and Supplemental Data 2). Only 1 drug, namely amisulpride, which is known as an antagonist of DRD2, DRD3, and HTR7, was predicted to commonly reverse the disease signature and mimic the infliximab effect on arthritogenic SFs (Supplemental Figure 1C). Supplemental Figure 1D presents the 50 top candidate perturbations that were proposed by L1000CDS² (14) search engine, after being interrogated with the input gene lists (Supplemental Data 3). We further observed that small molecules, such as artesunate, an antimalarial agent (16, 17), and vorinostat, a histone de-acetylase inhibitor (18), which have been suggested to deactivate arthritic fibroblasts, were also predicted to reverse our SF pathogenic expression profile, providing a further validation for the use of the L1000CDS^2^ search engine. We have therefore selected amisulpride as the most promising candidate for further validations.

### Validation of amisulpride as a modifier of hTNFtg SF activation.

**Figure 1:**
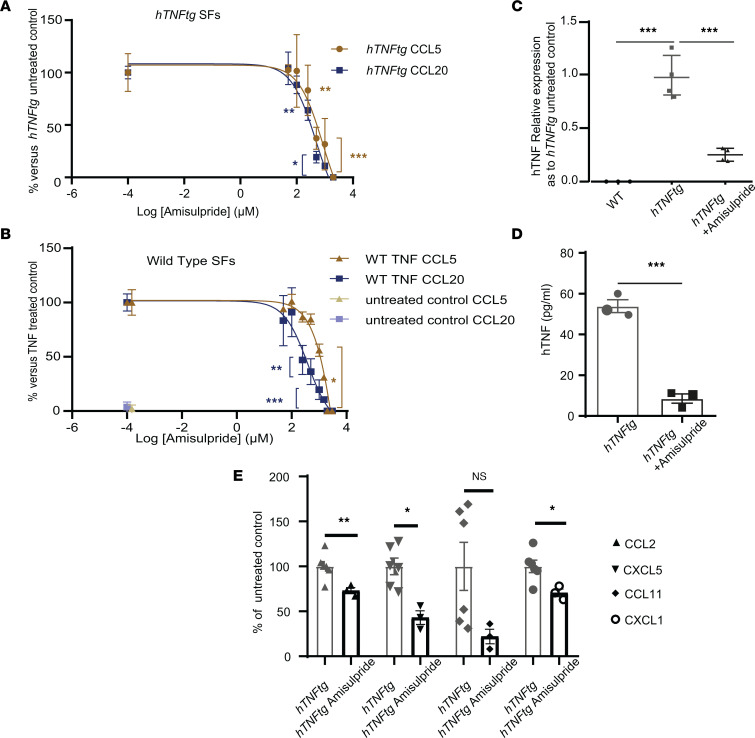
The antiinflammatory activity of amisulpride. (**A**) CCL5 and CCL20 detection in supernatants of *hTNFtg* SFs treated ex vivo with the indicated concentrations of amisulpride for 48 hours when compared with the *hTNFtg* vehicle-treated control (*n* = 3). (**B**) CCL5 and CCL20 detection in supernatants of WT SFs stimulated with 10 ng/mL hTNF, treated ex vivo with the indicated concentrations of amisulpride for 48 hours, when compared with the hTNF vehicle-treated control (*n* = 3). (**C**) Quantitative PCR (qPCR) analysis of *hTNF* gene expression in *hTNFtg* SFs treated with 500 μM amisulpride for 48 hours when compared with the *hTNFtg* vehicle-treated control (*n* = 4). (**D**) hTNF quantification in the supernatants of *hTNFtg* SFs when treated ex vivo with 500 μM amisulpride for 48 hours when compared with the *hTNFtg* vehicle-treated control (*n* = 3). (**E**) CCL2 (MCP1), CXCL5 (LIX), CCL11, and CXCL1 measured by Legendplex panel (BioLegend) on *hTNFtg* SFs treated with 500 μM amisulpride for 48 hours in comparison with the vehicle-treated control. TARC (CCL17), MIP-1β (CCL4), BLC (CXCL13), and MDC (CCL22) measured by Legendplex panel were not detected in the supernatants (*n* = 3–6) (* *P* value < 0.05; ** *P* value < 0.01; *** *P* value ≤ 0.0001; all data are shown as mean ± SEM; statistics for **A**, **B**, **D**, and **E** are performed using Student’s 2-tailed *t* test; statistics for **C** are performed using 1-way ANOVA, followed by Dunnett’s multiple comparisons test).

To assess the antiinflammatory properties of amisulpride on *hTNFtg* SFs, we measured its effect on the production of CCL5/RANTES and CCL20/macrophage inflammatory protein 3 (MIP-3). These 2 chemokines served as markers of pro-inflammatory activation of arthritic SFs, as well as indicators of SF deactivation following treatment with infliximab (Supplemental Data 1 and 2). Interestingly, amisulpride effectively downregulated the production of these chemokines in *hTNFtg* SF supernatants in a dose-dependent manner (Figure 1A). A similar effect of amisulpride on CCL5 and CCL20 production could also be observed in WT SFs exogenously stimulated by TNF (Figure 1B). These data indicate that amisulpride interferes with the pro-inflammatory activation of SFs, upon both chronic and acute TNF stimulation. The concentration representing the average IC_50_ value of amisulpride efficacy in downregulating the 2 chemokines was measured to be at the level of 500 μM. Of note, all drug concentrations used (50–1,000 μM) were nontoxic according to a crystal violet assay (Supplemental Figure 2A). Moreover, production of the monocyte chemoattractant protein-1 CCL2, the angiogenic chemokine CXCL5, and the neutrophil chemoattractant CXCL1 was also found to be reduced in the supernatants of *hTNFtg* SFs treated with 500 μM of amisulpride, indicating that this drug covers a wide spectrum of antiinflammatory properties (Figure 1E). Notably, amisulpride also reduced both the transcription and the production of soluble hTNF by *hTNFtg* SFs, regulating thereby the main driver of the arthritogenic activation of SFs (Figure 1, C and D).

**Figure 2:**
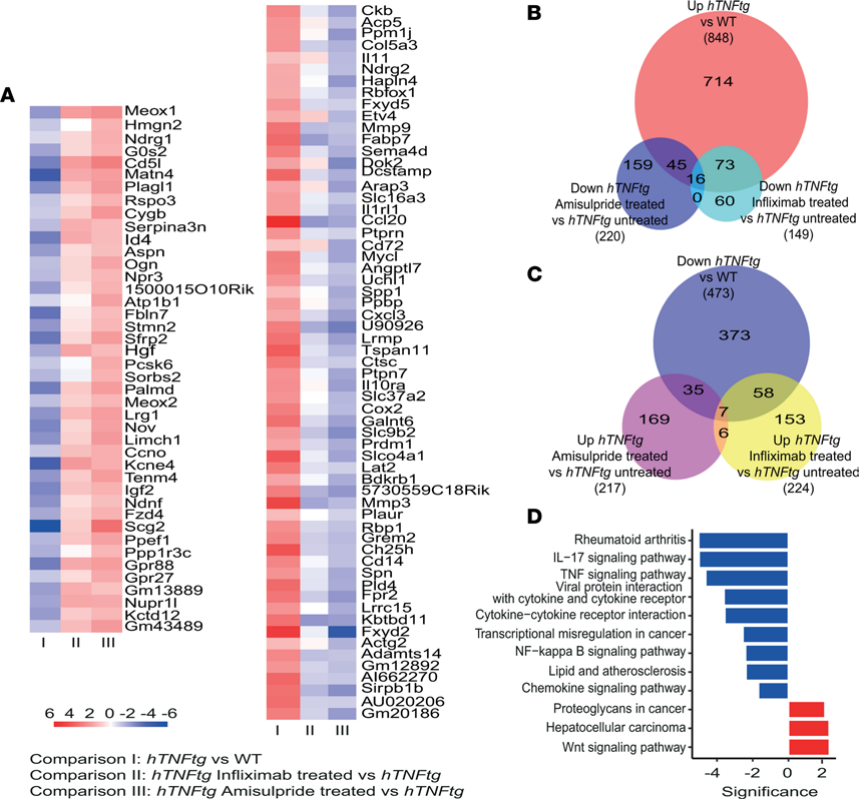
Gene expression signature of *hTNFtg* SFs treated with amisulpride. (**A**) Heatmap of the 103 genes identified to be deregulated in *hTNFtg* versus WT SFs and restored upon amisulpride treatment (*n* = 3). Left panel describes genes that are downregulated in *hTNFtg* versus WT SFs and are upregulated significantly upon amisulpride treatment. Right panel describes genes that are upregulated in *hTNFtg* versus WT SFs and are downregulated significantly upon amisulpride treatment. (**B**) Venn diagram presenting the genes being upregulated in *hTNFtg* versus WT SFs while being downregulated upon amisulpride/infliximab treatment (*n* = 3). (**C**) Venn diagram presenting the genes being downregulated in *hTNFtg* versus WT SFs while being upregulated upon amisulpride/infliximab treatment (*n* = 3). (**D**) KEGG pathway analysis of the 103 genes described in **A** (*n* = 3).

We further validated the effect of amisulpride on *hTNFtg* SFs by comparing the expression profile of *hTNFtg* SFs treated for 48 hours with 500 μM amisulpride with that of untreated *hTNFtg* SFs (Supplemental Data 4). Amisulpride affected 437 of the 1,321 differentially expressed genes in *hTNFtg* versus WT SFs (Supplemental Figure 2, B and C). Interestingly, amisulpride significantly downregulated 61 genes that were overexpressed in *hTNFtg* SFs (Figure 2, A and B). Several pro-inflammatory chemokines and metalloproteinases (MMPs) were verified to be significantly reduced by the treatment (Figure 2A). Kyoto Encyclopedia of Genes and Genomes (KEGG) pathway analysis of the deregulated genes revealed RA pathogenesis and TNF signaling among the top enriched terms (Figure 2D). Upregulated expression of genes known to be important in joint inflammation and cartilage and bone destruction, such as *Mmp3*, *Cxcl3*, and *Cox2*, was observed in *hTNFtg* SFs and found to be decreased following amisulpride treatment of arthritic SFs (Supplemental Figure 2D).

Notably, amisulpride treatment resulted in the overexpression of 42 genes that were found to be downregulated in *hTNFtg* versus WT SFs (Figure 2C). Pathway analysis of these 42 genes (Figure 2D) highlighted Wnt signaling as the top enriched pathway. Interestingly, Wnt signaling is known to be deregulated both in human RA and in animal models of disease (19, 20), while it has been suggested to play a protective role in *hTNFtg* mice (21). Moreover, expression levels of 23 out of the total 103 up- or downregulated genes were commonly regulated by amisulpride and infliximab treatment (Figure 2, A–C). Finally, amisulpride was able to effectively inhibit TNF-induced cytotoxicity of L929 cells (22) (Supplemental Figure 2E), providing further evidence on the potential interference of the drug with the TNF signaling pathway and rendering it as a promising candidate for further in vivo validations.

### Amisulpride alleviates acute and chronic inflammation in vivo.

Inhibitors of dopamine receptors, other than amisulpride, have been shown to decrease the LPS-induced plasma levels of proinflammatory cytokines and to inhibit the LPS-induced NO production by peritoneal macrophages (23, 24). To confirm that amisulpride shares similar in vivo functions, we used the acute LPS model of endotoxemia as well as the *hTNFtg* and *TNF*^ΔARE^ animal models of chronic inflammatory arthritis.

Indeed, administration of amisulpride downregulated significantly the elevated serum levels of mouse TNF and IL-6 detected 1.5 hours after induction of LPS in a dose-dependent manner (Supplemental Figure 3A).

Considering the inhibitory activity of amisulpride on the TNF signalling pathway in fibroblasts ex vivo, the therapeutic potential of the drug was further evaluated in vivo, in the *hTNFtg* fibroblast-dependent model (2). Amisulpride was administered orally twice per day with a prophylactic regime (before the onset of the disease until the established state, from the third to the eighth week of age). The selected dose of amisulpride (20 mg/kg) was based on the known toxicity data of the drug and the relevant human dose prescribed for its original use in depressive disorders (25, 26).

**Figure 3:**
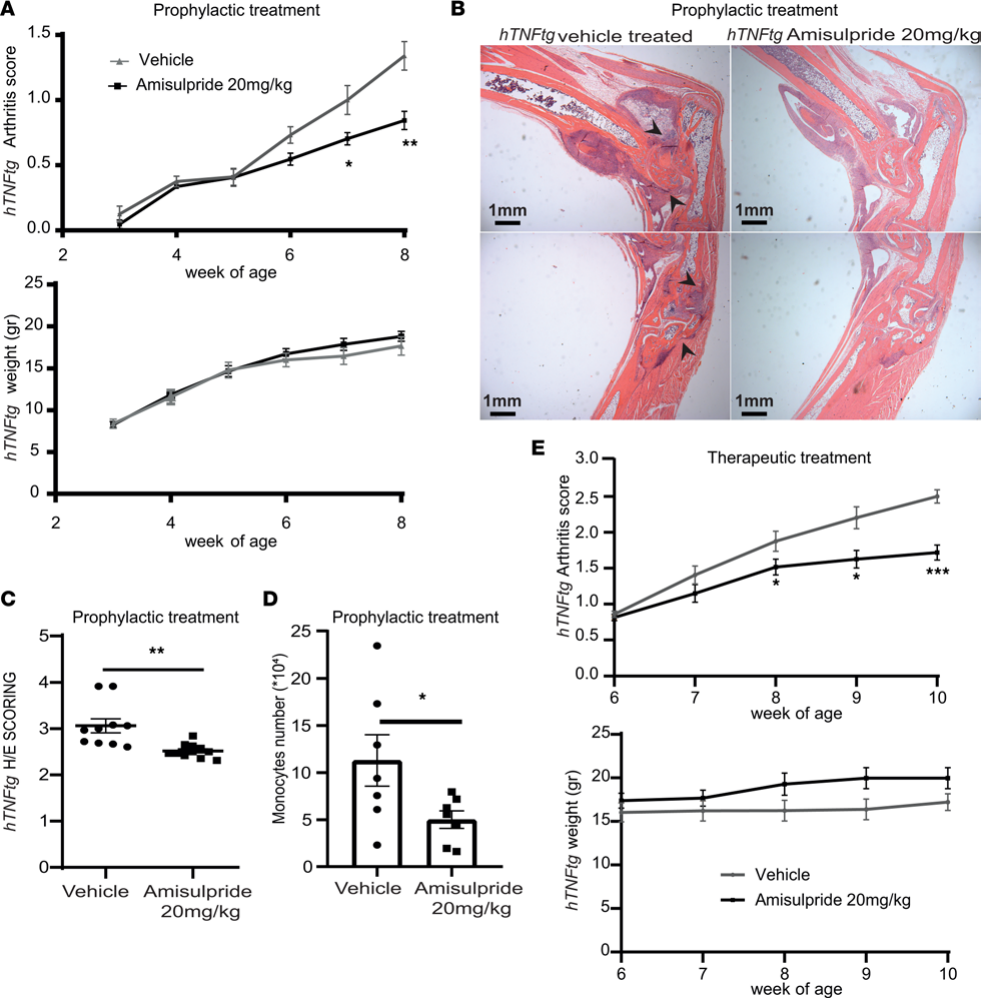
In vivo effect of amisulpride in the *hTNFtg* polyarthritis model. (**A**) Arthritis clinical score and weight measurement of *hTNFtg* mice treated prophylactically with 20 mg/kg amisulpride by oral gavage (week 3–week 8) when compared with the vehicle-treated controls (*n* = 10). (**B**) Representative histological images of H/E-stained paraffin sections of joints of *hTNFtg* mice treated prophylactically with 20 mg/kg amisulpride by oral gavage (week 3–week 8) when compared with the vehicle-treated controls (original magnification 2×, scale bar = 1 mm). The sections in the top and bottom row show ankle and metatarsal field of the same representative section, and black arrows indicate examples of regions of interest. (**C**) Synovitis scoring of H/E-stained paraffin sections of ankle joints of *hTNFtg* mice treated prophylactically with 20 mg/kg amisulpride by oral gavage (week 3–week 8) when compared with the vehicle-treated controls (*n* = 10). (**D**) Monocyte numbers in FACS-based immune infiltration analysis of isolated ankle joints of *hTNFtg* mice treated prophylactically with 20 mg/kg amisulpride by oral gavage (week 3–week 8) when compared with the vehicle-treated controls (*n* = 7). (**E**) Arthritis clinical score and weight measurement of *hTNFtg* mice treated therapeutically with 20 mg/kg amisulpride by oral gavage (week 6–week 10) when compared with the vehicle-treated controls (*n* = 8) (* *P* value < 0.05; ** *P* value < 0.01; *** *P* value ≤ 0.0001; all data are shown as mean ± SEM, statistics are performed using Student’s 2-tailed *t* test).

Amisulpride decreased the clinical arthritis score of *hTNFtg* mice (Figure 3A), also decreasing significantly the synovitis score in H/E-stained histological sections (Figure 3, B and C). Intriguingly, the significant antiarthritic activity of amisulpride was further associated with a trend over reduction of the osteoclast numbers in the ankle joints, while its effect on the cartilage destruction score was not significantly evident (Supplemental Figure 4, A–D). Weekly body weight assessment and gross behavioral effect observation verified the well-tolerated safety profile of the drug (Figure 3A). Of note, no significant differences in the serum levels of hTNF could be observed between treated and untreated mice (Supplemental Figure 4E), indicating that, in the vivo setting, the improved clinical score is not associated with a systemic downregulation of TNF production.

FACS-based quantification of the infiltrating macrophages, monocytes, neutrophils, eosinophils, dendritic cells, B cells, CD4^+^ T cells, and CD8^+^ T cells, as well as the CD90^–^ lining and CD90^+^ sublining SFs, which are found to be increased in the ankle joints of the diseased *hTNFtg* mice when compared with the WT controls (27), indicated that prophylactic amisulpride treatment attenuated mainly the number of monocytes when compared with the vehicle-treated controls (Figure 3D and Supplemental Figure 5). This could be associated with the decreased CCL2 levels detected on the *hTNFtg* SFs when treated with the drug (Figure 1E). Notably, when amisulpride was administered to *hTNFtg* mice therapeutically (i.e., mice treated with 20 mg/kg orally after the establishment of disease, from 6 to 10 weeks of age), the amelioration of clinical score was associated with reduction of both the synovitis and the number of osteoclasts in the ankle joints (Figure 3E and Supplemental Figure 4, F–I).

To assess the activity of amisulpride in yet another model of chronic polyarthritis that does not depend on TNF production by the synovial fibroblasts, we employed the *TNF*^ΔARE^ mouse model that was shown to depend on TNF production by hematopoietic cells (5). Notably, prophylactic administration of amisulpride in this model resulted in the alleviation of both clinical and histological scores associated with significant reductions in all pathological indicators, including synovitis, bone erosions, and cartilage destruction (Supplemental Figure 6, A–C).

Taken together, these results show that depending on context (type of preclinical model, pathogenic mechanism, and clinical stage of drug administration), amisulpride has the potential to interfere with both the inflammatory and the destructive pathological signs of arthritis progression.

### The effect of amisulpride on SFs is mediated neither via its known targets nor through inhibition of TNF-TNFRI interactions.

**Figure 4:**
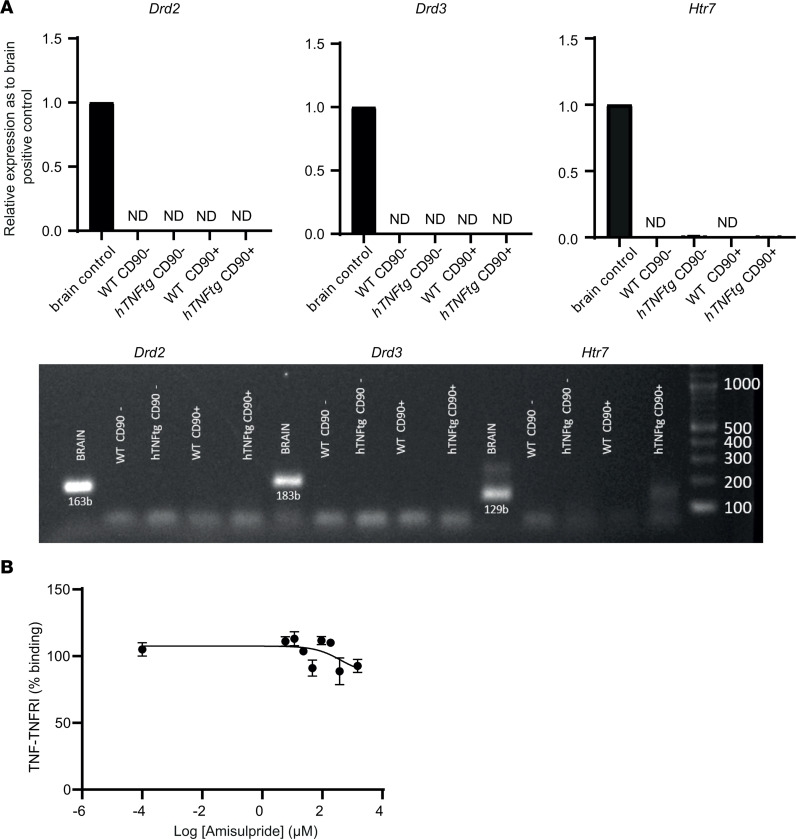
Amisulpride’s effect on joint fibroblasts is mediated neither by its known targets nor through TNF-TNFRI binding inhibition. (**A**) qPCR analysis for quantification of *Drd2*/*Drd3*/*Htr7* expression on CD31^–^CD45^–^PDPN^+^CD90^–^ lining or CD31^–^CD45^–^PDPN^+^CD90^+^ sublining SFs isolated from ankle joints of WT or *hTNFtg* animals. Brain sample was used as a positive control (*n* = 5). ND, not detected. (**B**) TNF-TNFRI binding upon amisulpride in a dose-dependent manner (*n* = 3).

We next investigated the molecular mechanism of amisulpride function. We first assessed whether amisulpride mediates its effects through its known targets, DRD2/DRD3/HTR7 receptors. Although B and T lymphocytes, macrophages, and dendritic cells have been shown to express dopamine receptors (28), their expression in RA FLS has remained controversial (29, 30). In the present study, *Drd2*/*Drd3*/*Htr7* gene expression was detectable neither in the cultured nor in the freshly isolated CD90^–^ or CD90^+^ *hTNFtg* SFs (8) (Figure 4A). It therefore seems that in the *hTNFtg* model, the deactivating effect of amisulpride on SFs is mediated by targeting molecules other than its known dopamine or serotonin receptors. Additionally, the ability of the drug to directly interrupt the interaction of TNF with its receptor TNFRI was quantified. As shown in Figure 4B, amisulpride did not suppress the binding of TNF to its main receptor TNFRI, indicating that the overall anti-TNF effect of the drug is not mediated through inhibition of TNF-TNFRI binding. In conclusion, these results suggest that the activity of amisulpride on *hTNFtg* SFs is not mediated by the main known targets of the drug (DRD2, DRD3, and HTR7), while its effect on TNF signaling is not due to the interruption of binding of TNF to TNFRI.

### Chemoproteomic identification of potential molecular targets of amisulpride on arthritic SFs.

**Figure 5:**
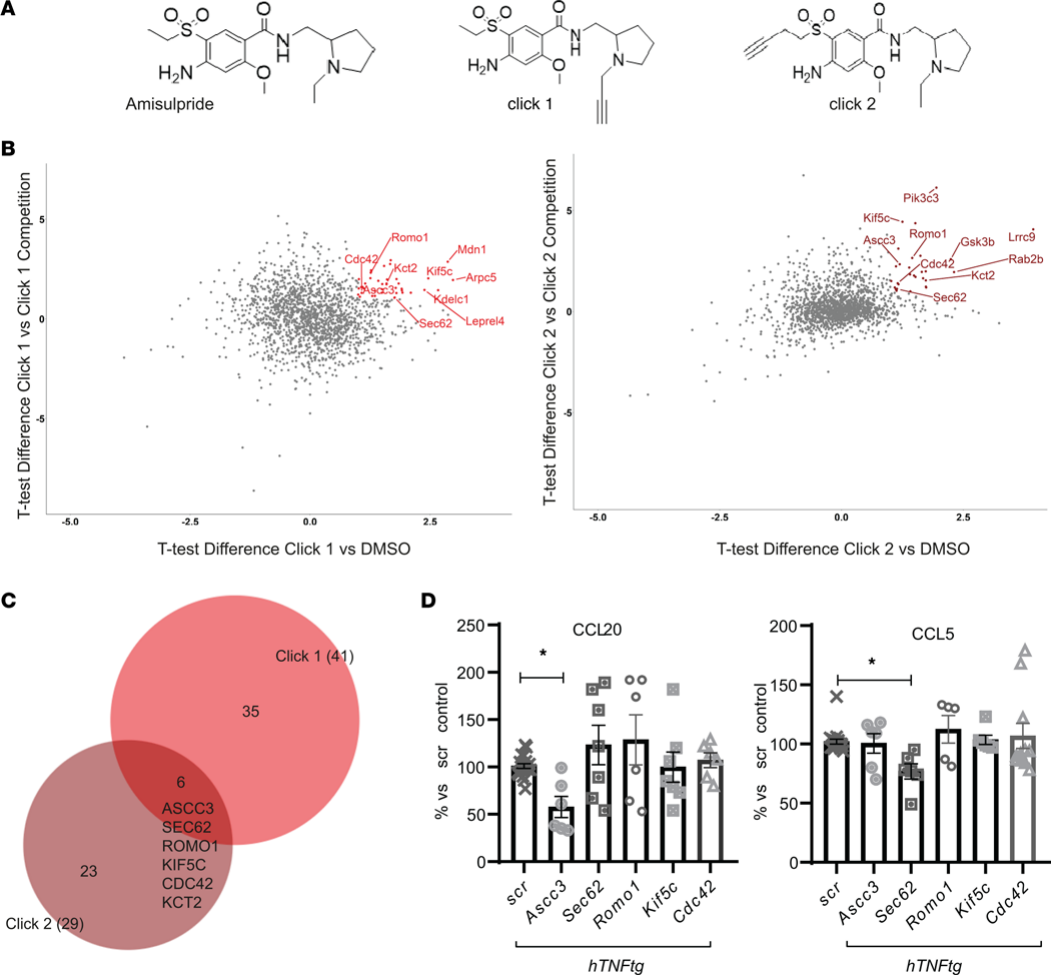
Amisulpride targets’ identification on *hTNFtg* SFs. (**A**) Structure of the amisulpride and the synthesized click compounds, based on amisulpride chemical form, bearing an alkyne in a different position. (**B**) Volcano plots with highlighted candidates, combining differences (Student’s 2-tailed *t* test difference > 1) for proteins identified in each active probe sample versus the DMSO or the respective competition control (*n* = 3). (**C**) Venn diagram of the proteins identified in volcano graphs of **B**, resulting in 6 common candidates targeted by both click 1 and click 2. (**D**) CCL20 and CCL5 quantification in supernatants derived from *hTNFtg* SFs upon shRNA-mediated silencing of *Ascc3*, *Sec62*, *Romo2*, *Kif5c*, and *Cdc42* when compared with the scramble-treated sample (*n* = 6–20) (* *P* value < 0.05; all data are shown as mean ± SEM; statistics are performed using 1-way ANOVA, followed by Dunnett’s multiple comparisons test). ASCC3, activating signal co-integrator 1 complex subunit 3; SEC62, translocation protein SEC62; CDC42, cell division cycle 42; KIF5C, kinesin heavy chain isoform 5C; ROMO1, ROS modulator 1; KCT2, keratinocyte-associated transmembrane protein 2.

To identify potentially direct targets of amisulpride on SFs, we designed and synthesized 2 bioactive chemical probes, which structurally resemble amisulpride (click compounds), while containing a click handle in a different position (triple bond in Figure 5A). The click compounds can ligate to biotin and pull down the potential target proteins after being bound to streptavidin beads, thus enabling target identification through liquid chromatography-tandem mass spectrometry analysis (31, 32). We synthesized 2 click probes in order to exclude as many artifacts as possible and to end up with a low number of potential protein targets for validation. In the first click probe (click 1, Figure 5A), the alkyne was incorporated into amisulpride as substituent of the pyrrolidine group, while in the second click probe, the alkyne was included as substituent of the sulfone group (click 2, Figure 5A). The optimized synthetic route followed for the synthesis of the 2 click probes is displayed in Supplemental Figure 7, A and B. Supplemental Figure 8 includes the characterization of the synthesized amisulpride and its click derivatives (click 1 and click 2) by ^1^H NMR and mass spectrometry.

To verify that both click compounds retain their bioactivity, we repeated the CCL5 and CCL20 quantification in *hTNFtg* SFs treated with each individual probe. Both click analogs exhibited an effect similar to or even better (Supplemental Figure 7C) than amisulpride (Figure 1A).

Hence, the *hTNFtg* SFs were treated with each click derivative (25 μM), while 2 negative controls were used. The first control included *hTNFtg* SFs treated with vehicle (DMSO, no probe). The second control (competition) included *hTNFtg* SFs treated with each click derivative (25 μM) plus an excess concentration (10×, 250 μM) of amisulpride, which would compete off each probe (click 1/click 2) and potentially occupy the target binding sites. The target proteins were identified by mass spectrometry after being conjugated with a biotin tag and pulled down on streptavidin beads. The mass spectrometry results were further analyzed by (a) plotting the protein enrichment by each probe (click 1/click 2) compared with vehicle (DMSO) and (b) plotting the proteins enriched by click 1/click 2 compared with the competitor (amisulpride) (Figure 5B and Supplemental Data 5). Combining differences (Student’s *t* test difference > 1) in proteins identified in both active probe samples but not in the 2 negative controls (DMSO/amisulpride) resulted in a shortlist of potential targets (Figure 5C). The 6 common candidates identified were ASCC3, SEC62, ROMO1, KIF5C, CDC42, and KCT2 (Figure 5C). KCT2 was then excluded from further analysis, as it is a protein expressed mainly by keratinocytes, indicating that it was probably a contaminant protein in the experiment.

In conclusion, we found that amisulpride possibly regulates the activation of *hTNFtg* SFs by binding to ASCC3, SEC62, ROMO1, KIF5C, and/or CDC42.

### Validation of potential molecular targets of amisulpride.

The identified targets seemed potentially capable of ameliorating *hTNFtg* SFs’ pathogenicity. *Ascc3* encodes a 3′–5′ DNA helicase, and in cell lines loss of *Ascc3* leads to reduced cell proliferation (33). KIF5C is located in microtubules, where among other proteins, it has been found to be necessary for the transport of N-cadherin between the Golgi and the plasma membrane, facilitating cell-to-cell adhesion in fibroblasts (34). CDC42 has also been associated with adherence of fibroblasts as it belongs to a subfamily of Rho GTPases (35), while it has been previously linked to compounds targeting the HTR7 receptor (36). ROMO1 is localized in the mitochondria, being responsible for TNF-induced ROS production (37). Last, SEC62 is located in the endoplasmic reticulum (ER) and is responsible, as a member of the SEC61/62/63 complex, for proteins’ translocation in the ER and for cell calcium regulation (38).

To verify further the role of the 5 proteins identified as potential molecular targets of amisulpride in the *hTNFtg* arthritic SFs, we designed shRNAs to silence the expression of each one of their corresponding genes using the Lenti-X Lentiviral expression system. After verifying the significant reduction of the relevant mRNA (Supplemental Figure 7D) and the viability of the cells (Supplemental Figure 7E) upon each transfection, we checked which of the potential targets could successfully downregulate both CCL5 and CCL20, resulting in an antiinflammatory effect similar to amisulpride (Figure 5D). Of note, deletion of *Ascc3* and *Sec62* significantly reduced pathogenic chemokine levels (CCL20 and CCL5, respectively). This result indicates that the amelioration of *hTNFtg* SFs’ inflammatory activation offered by amisulpride is potentially through targeting of both ASCC3 and SEC62.

The antiinflammatory role of ASCC3 and SEC62 targeting could also be correlated with the effect of amisulpride on *hTNFtg* SFs gene signature (Figure 2D). Analysis of the expression profile of the treated *hTNFtg* SFs revealed the TNF/NF-κB pathway and chemokine signaling as major cascades regulated by the drug, verifying that the molecular targets of the drug may play a role in the response to inflammatory signals.

In conclusion, 5 potential amisulpride targets on *hTNFtg* SFs were identified, and we propose herein that the antiinflammatory profile of the drug is supported by the dual targeting of *Assc3* and *Sec62*.

### Amisulpride influences pathways known to be implicated in the arthritogenic activation of SFs.

To gain a deeper insight into the cellular signaling affected by the drug, we employed phosphoproteomic profiling of treated SFs (39). hTNF-induced WT SFs rather than *hTNFtg* SFs were used to achieve synchronization in the context of TNF stimulation and signaling. Three time points of hTNF induction of SFs were used and compared with cells pretreated with amisulpride 1 hour before being stimulated with hTNF at the respective time points (5, 15, 30 minutes).

Initially, all amisulpride-treated samples were clustered together and were compared versus the untreated ones. Supplemental Figure 9A displays a volcano plot that integrates the deregulated phosphosites in treated samples when compared with the respective untreated controls (Supplemental Data 6). Notably, hierarchical clustering revealed clearly distinguishable groups of phospho-regulations that were dependent on the presence of the inhibitor (Supplemental Figure 9B).

**Figure 6:**
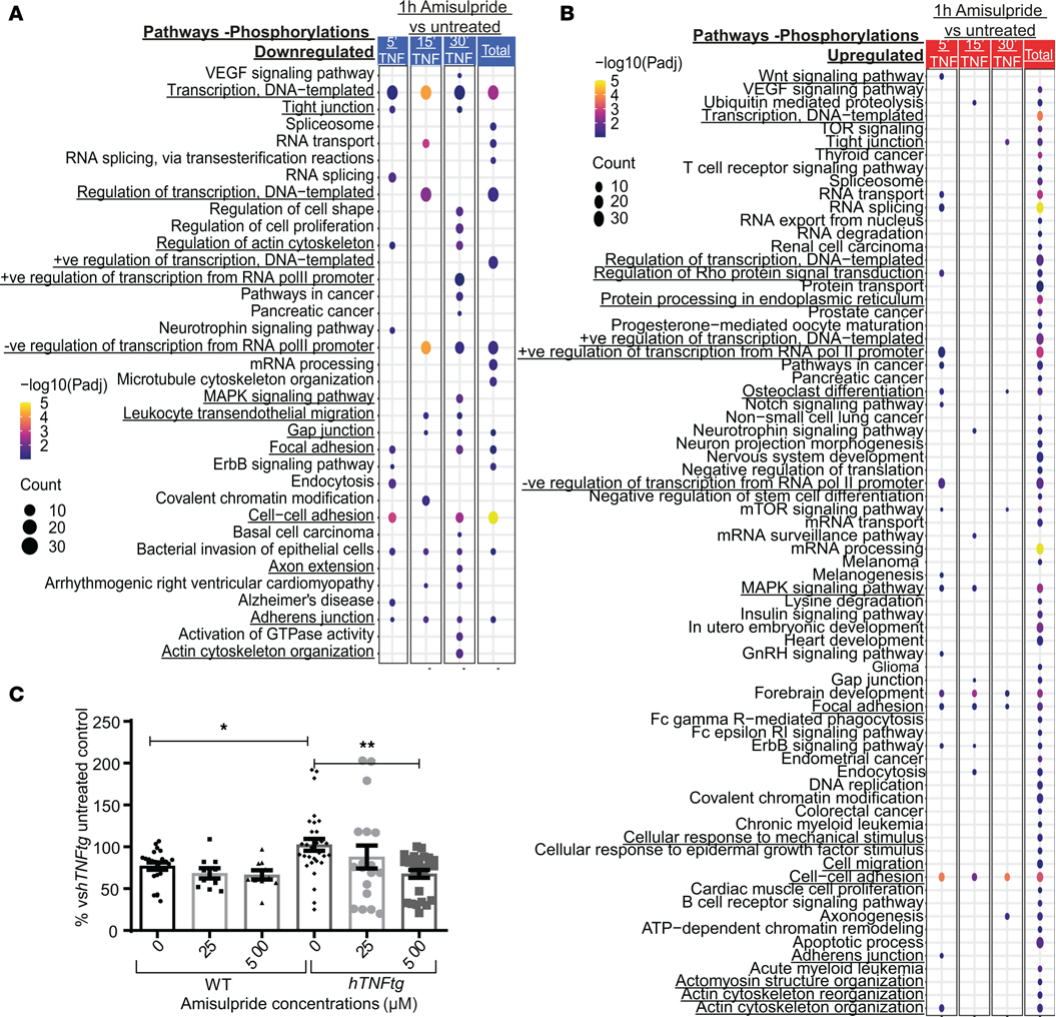
Phosphoproteomics analysis of intraarticular SFs treated with amisulpride reveals pathways that are regulated upon treatment. KEGG pathways and Gene Ontology term enrichment analysis for (**A**) downregulated and (**B**) upregulated phosphorylations upon pretreatment with 500 μM amisulpride for 1 hour and stimulation with hTNF for 5, 15, and 30 minutes when compared with the untreated hTNF-stimulated respective controls (*n* = 3). (**C**) Adherence assay of WT and activated *hTNFtg* SFs upon different amisulpride concentrations (*n* = 18–30 wells) (* *P* value < 0.05; ** *P* value < 0.01; all data are shown as mean ± SEM; statistics are performed using 1-way ANOVA, followed by Dunnett’s multiple comparisons test).

Moreover, phosphosites were analyzed independently for the 3 time points of hTNF stimulation (volcano plots in Supplemental Figure 9C and Supplemental Data 7). Both the KEGG pathways and the Gene Ontology terms enriched in all comparisons were analyzed (Figure 6, A and B).

Most of the phosphosites were upregulated upon inhibitor treatment, highlighting cell adherence, focal adhesion, and MAPK signaling as implicated processes, pathways that are known to play a role in pathogenic fibroblast activation (Figure 6B). Focusing on the downregulated phospho-changes, similar enriched processes were found to be involved, including cell-to-cell adhesion, focal adhesion, and adherence junction, as well as regulation of transcription (Figure 6A).

Notably, adhesion is known to be enhanced in pathogenic *hTNFtg* fibroblasts when compared with WT controls (40), and amisulpride was found to significantly reduce ex vivo cell adherence, validating the pathways proposed to be regulated by the drug in the phosphoproteome analysis (Figure 6C).

Interestingly, some of the identified protein targets of the drug (Figure 5C) can be highly connected with the pathways regulated upon amisulpride treatment on SFs. Specifically, the phosphorylation of SEC62 (at Ser341), validated as one of amisulpride’s targets on SFs, seemed to be upregulated upon treatment, indicating that probably the ER translocation pathway was affected by the drug (Figure 6B). In addition, another potential drug target, CDC42, is a Rho GTPase known to regulate filopodia formation and adherence of fibroblasts (35, 41). Importantly, Rho protein signal transduction, cell-to-cell adhesion, and actin cytoskeleton organization were found to be regulated too by amisulpride, as shown in Figure 6, A and B. Moreover, CDC42EP1 (MSE55), a CDC42 binding protein that regulates cytoskeleton dynamics, was dephosphorylated (at S371 and S207) upon amisulpride treatment, underlining further cell-to-cell adhesion and actin cytoskeleton dynamics alteration upon treatment (Figure 6, A and B). Finally, ASCC3, as an RNA helicase, is implicated in transcription regulation, one of the top pathways identified in the phosphoproteome analysis (Figure 6, A and B).

Together, these results show that amelioration of *hTNFtg* arthritis by amisulpride is mediated mainly by targeting SF-specific inflammatory and adhesive signaling pathways, and, at least in part, via the above-described molecular targets.

## Discussion

In this study, following a gene expression–based repurposing approach, we identified amisulpride as a modifier of arthritic fibroblast activation and fibroblast-dependent *hTNFtg* polyarthritis. Specifically, we have shown elevated chemokines and adhesive properties of arthritic fibroblasts to be remarkably downregulated ex vivo after amisulpride treatment and *hTNFtg* polyarthritis to be alleviated after prophylactic but importantly also upon therapeutic administration of the drug. Amisulpride is a DRD2/DRD3/HTR7 inhibitor that has been widely used in high doses to treat schizophrenia and in low doses for the treatment of depressive disorders (42, 43). However, we show here that its activity on *hTNFtg* fibroblasts is not mediated through these receptors. Interestingly, chemoproteomics analyses in SFs identified potential drug targets, namely ASCC3, SEC62, KIF5C, ROMO1, and CDC42. shRNA-mediated silencing of ASCC3 and SEC62 in SFs showed antiinflammatory activities similar to those exerted by amisulpride. Future detailed target characterization experiments should reveal further amisulpride targets and pathways relevant to SFs.

Dopamine receptor inhibitors, apart from their main function in neuronal signal transduction, have been previously implicated in immune regulation (44) and are reported to suppress pro-inflammatory cytokine production in LPS-induced macrophages (45) and in sepsis models in vivo (46), as well as to affect experimentally induced animal models of RA (47, 48). Similarly, we have validated here the ameliorating effect of amisulpride in the acute LPS-induced model of sepsis and, importantly, in the *TNF*^ΔΑRE^ mouse model of chronic arthritis, where the pathogenic source of TNF lies with the bone marrow compartment (5). This study indicates that in addition to the known antiinflammatory activity of amisulpride in myeloid and immune cells, the drug demonstrates a potentially novel antiinflammatory, antiarthritogenic function on SFs, which is dopamine receptor independent and works via a potentially novel set of target proteins, broadening the translational value of amisulpride for the treatment of chronic inflammatory arthritis.

Thus, the probably new activity of amisulpride in the *hTNFtg* mouse model could be synergistic. It could be ascribed to DRD2, DRD3, and HTR7 antagonism in myeloid/immune cells that are known to express these receptors, and as we show here, it could be additionally functioning as inhibitor of the newly identified targets on arthritogenic fibroblasts. Two of these targets, namely ASCC3 and SEC62, have been validated in our study to mediate inflammatory effects similar to those modified by amisulpride. This dual role underlines the strong potential of repurposing this specific drug for RA treatment. Considering that de novo identification of RA therapeutics is both costly and time consuming, drug repositioning presents as a tractable alternative, since essential issues such as bioavailability, toxicity, and manufacturing routes of the already-tested drugs are known and established (13).

Considering the potentially new SF-specific amisulpride targets we have validated in this study, it is interesting to note that they cover a range of SF activation characteristics, such as proliferation and migration, transcriptional and translational activation of inflammatory genes, homeostatic ER stress, and calcium responses. ASCC3 is the largest subunit of the ASC1 complex, where it functions as a 3′–5′ DNA helicase, together with ASCC1, ASCC2, and ASC1/TRIP4 (33, 49). Inhibitors targeting DNA/RNA helicases have been proposed to regulate viral and bacterial responses as well as cancer cell proliferation (33, 50). Ascc3 is also considered to participate in DNA repair pathways, as it prepares single-stranded DNA for AlkBH3 to mediate de-alkylation repair (33) and it resolves stalled ribosomes (51, 52). Moreover, the ASC1 complex has been involved in transcriptional regulation, regulating alone, or through the transcription integrators SRC-1 and CBP-p300, several transcription factors such as CREB, STATs, AP-1, and NF-κB (49, 53). ASCC3 involvement in the regulation of these inflammation-related transcription factors (54) can be further substantiated by the findings of the present study, where *Ascc3* downregulation was able to cause a marked reduction of the pro-inflammatory CCL20 secretion in TNF-induced activated SFs.

The translocation protein SEC62, on the other hand, is a part of the dimeric SEC62/SEC63 complex, which along with Sec61 is located in the membrane of the ER, facilitating translocation of nascent polypeptides into the ER and cell calcium homeostasis (38, 55, 56). Additionally, SEC62 has been reported to play a crucial role in successful ER stress response, as it favors the release of the accumulated ER chaperones driving cell physiological homeostasis (56). Notably, SEC62 has been reported as oncogenic, being overexpressed in a variety of tumors (57), supporting tumor metastasis, which can be attributed either to the deregulated translocation of migration-related precursor proteins to the ER or to the inhibition of Ca^2+^ homeostasis, along with the attributed ER stress tolerance (38). In particular, no SEC62-specific inhibitors have been reported to date, but inhibition of Sec62 function by antagonizing cellular Ca^2+^ homeostasis by CaM antagonists has been proved to mimic *SEC62* deletion in vitro by inhibiting migration and proliferation of human tumor cells (57, 58). Targeting of SEC62 could also be beneficial in RA, since SEC62 has been found to be induced in a human RA synovial tissue study, especially in a patient cohort that is characterized by expansion of FLS and not by predominance of the myeloid/lymphoid compartment before receiving any treatment (59). This subgroup of patients can probably be simulated by *hTNFtg* mice, where TNF signaling specifically in fibroblasts is necessary and sufficient to initiate disease (5, 6).

Considering that amisulpride is currently used in the market as an antipsychotic drug, it is important to note that almost 19% of patients with RA develop depression, a percentage much higher than the average in the general population (60). A common association of RA with its comorbid depression could be the fatigue and the pain that patients experience (61, 62), which can also activate immune responses, causing further dysthymia pathology (63–65). Of note, anti-TNF biologics have been proposed to alleviate both RA and depression symptoms (62); however, their prescription is approved only in severe cases of RA, and not all patients respond to them, thereby necessitating the frequent prescription of additional antidepressants (66). Consequently, patients experiencing RA and comorbid depression may be highly benefited by the use of amisulpride. Importantly, clinical use of several dopamine receptor antagonists in patients with schizophrenia has been associated with a much lower incidence of RA compared with the incidence in the general population (67). Accordingly, studying patients with RA under amisulpride treatment, prescribed for their depression symptoms, for possible beneficial clinical and histological RA outcome might provide valuable knowledge and clinical validation for the antiarthritic effects of the drug.

The *hTNFtg* animal model has been proved to closely simulate human disease, sharing common SF subpopulations and gene expression profiles (2, 7, 10). Notably, here, the oral administration of a small molecule, amisulpride, caused a significant amelioration of *hTNFtg* arthritis not only prophylactically but also therapeutically. Amisulpride has been identified as an inhibitor of pro-inflammatory cytokine, chemokine, and MMP production, as well as of adhesion of activated SFs ex vivo. However, the alleviation of polyarthritis in *hTNFtg* mice was associated mainly with the reduction of immune infiltration and bone destruction of the ankle joints, not with reductions in cartilage degradation. This could be due to context- and model-dependent development of the clinical phenotype, as notably, in *TNF*^ΔARE^ mice, all 3 histological manifestations were considerably affected. In conclusion, our studies strongly indicate that amisulpride could be repurposed for the treatment of RA and that it may also be used as a promising “lead” compound for the development of novel fibroblast-deactivating therapeutics.

## Methods

### Study design.

This study was performed in order to identify potential therapeutics against RA, by investigating if amisulpride can alleviate mouse chronic polyarthritis. Sample size of both the in vitro and in vivo experiments was calculated using power analysis. Ex vivo studies used primary murine ankle joint SFs of WT and *hTNFtg* mouse model of destructive polyarthritis as well as the L929 cell line. WT, *hTNFtg*, and *TNF*^ΔARE^ mice were used in the in vivo experiments. WT mice were originally purchased from Charles River Laboratories and The Jackson Laboratory. *hTNFtg* and *TNF*^ΔARE^ mice (2, 3) were provided in-house. The total number of replicates for the statistics calculation is noted in figure legends. Mouse welfare upon treatment (amisulpride or vehicle control) was checked every 24 hours in order to follow humane endpoints. All mice were observed for morbidity and euthanized when needed according to animal welfare. Mice were randomly assigned to the control and treatment groups, and clinical and histological scoring was performed under a blinded manner. No outliers were excluded apart from histology slides with no specific/high background staining. Each experiment, except RNA-Seq and proteomics studies, was performed at least 3 times, and data shown in the figures represent a representative experiment.

### SFs’ isolation.

Primary mouse SFs were isolated from ankle joints of 8-week-old CBA C57BL/6J, WT, or *hTNFtg* mice as previously described (68). Briefly, ankle joints were digested with Collagenase IV (Sigma-Aldrich, C5138), and at passage 1 a depletion of CD45^+^ cells was performed using a Biotin anti–mouse CD45 antibody (BioLegend, 103104) and Dynabeads Biotin Binder (Thermo Fisher Scientific 11047), according to the manufacturer’s protocol.

### Amisulpride.

For in vivo experiments, the commercially available Solian solution (Sanofi, 100 mg/mL) was used. For in vitro assays, synthesized amisulpride was used (100 mM stock in DMSO, further dilutions in 1× DMEM) (69).

### RNA-Seq analysis.

RNA-Seq was performed in 3 biological replicates of cultured WT/*hTNFtg* SFs treated with 1 μg/mL infliximab, 500 μM amisulpride, or vehicle for 48 hours. RNA extraction of cultured SFs was performed using the QIAGEN RNA Micro Kit (catalog 74004), according to the company’s guidelines. RNA extraction of 8-week-old *hTNFtg* mice’s back joints’ CD31^–^CD45^–^PDPN^+^ cells was performed using the Single Cell RNA Purification Kit (Norgen, 51800). All samples were quantified by a Bioanalyzer using the Agilent RNA 6000 Nano Kit reagents and protocol (Agilent Technologies). Only RNA samples with RNA integrity number greater than 7 proceeded to further analysis. The library preparation was performed according to the 3′ mRNA-Seq Library Prep Kit protocol for Ion Torrent (QuantSeq-LEXOGEN). The libraries quality was measured in a Bioanalyzer, using the DNA High Sensitivity Kit reagents (Agilent Technologies) according to manufacturer’s protocol, and they were equated at a concentration of 50 pM. Templating was performed in the Ion Proton Chef instrument, following the Ion PI IC200 Chef Kit (Thermo Fisher Scientific). Sequencing was performed in the Ion Proton System according to the Ion PI Sequencing 200 V3 Kit on Ion Proton PI V2 chips (Thermo Fisher Scientific).

The RNA-Seq FASTQ files obtained after Ion Proton sequencing were mapped using TopHat2 (70) with default settings and using additional transcript annotation data for the mm10 genome from Illumina iGenomes (https://support.illumina.com/sequencing/sequencing_software/igenome.html). According to the Ion Proton manufacturer’s recommendation, the reads that remained unmapped were submitted for a second round of mapping using Bowtie2 (71) against the mm10 genome with the “--very-sensitive” parameter activated and merged with the initial mappings. The raw bam files were summarized to a read counts table using the Bioconductor package GenomicRanges (72). Genes that had 0 counts were removed and the gene counts were normalized. Subsequently differential expression analysis was conducted using the Bioconductor package DESeq (73). Differentially regulated genes were concluded using an absolute log_2_ fold-change cutoff value of 1 and a *P* value cutoff of 0.05. The aforementioned analytical steps were performed through the Bioconductor package metaseqr (74). R packages (75) were used for generating volcano plots, heatmaps, bubble plots, and bar plots. Scaled Venn diagrams were created with the online tool BioVenn (76). Functional enrichment analysis was done with Enrichr online tool (77).

### LINCS1000CDS^2^ search.

The overlap of the deregulated genes from 8-week *hTNFtg* SFs versus WT controls and from previously published data at 3 disease points (3, 8, and 11 weeks old) (7) was used as an input to the search engine LINCS1000CDS^2^ (14), to identify perturbations that could potentially reverse the pathogenic gene expression profile (Supplemental Data 3, http://amp.pharm.mssm.edu/clustergrammer/l1000cds2/5b0ec39527c04c0700a07842). Additionally, the expression profile of 8-week *hTNFtg* SFs after a 48-hour treatment with 1 μg/mL infliximab (Janssen Biologics B.V) versus the *hTNFtg* untreated SFs was used as an input to identify compounds that could mimic the treated *hTNFtg* SFs’ signature (Supplemental Data 3, http://amp.pharm.mssm.edu/clustergrammer/l1000cds2/5b0ec77627c04c0700a07846). Specifically, for the reversed signature results, we used as an input 113 and 93 genes, which were found commonly up- and downregulated, respectively, in the comparison of *hTNFtg* versus WT untreated SFs in all stages of the disease. For the mimicked signature results, we used 224 and 149 genes, which were found up- and downregulated, respectively, in the comparison of 8-week *hTNFtg* SFs treated with infliximab versus 8-week *hTNFtg* untreated SFs (Supplemental Data 2). In each comparison of our data, we filtered the genes for *P* < 0.05 and log_2_ fold-change > 1 (upregulated genes) or *P* < 0.05 and log_2_ fold-change < –1 (downregulated genes). The output of both LINCS1000CDS^2^ result lists was used to construct the Venn diagram of treatments that followed both criteria, using Interactivenn (78).

### ELISAs.

SFs at passage 3 isolated from 8-week-old WT or *hTNFtg* ankle joints were starved overnight and were treated with different amisulpride concentrations. WT SFs were also stimulated with 10 ng/mL hTNF (Peprotech) or hTNF preincubated with amisulpride for 30 minutes. At 48 hours after, the cell culture supernatants were analyzed for the detection of CCL5 (DY478, DuoSet), CCL20 (DY760, DuoSet), hTNF (R&D Systems, DTA00D), or other chemokines (Mouse Legendplex Chemokine Panel, BioLegend, 740007) according to the manufacturer’s instructions. The toxicity of amisulpride was assessed using the crystal violet assay on SFs, as described elsewhere (79).

### Gene expression quantification.

qPCR analysis was performed using the Platinum SYBR Green qPCR SuperMix (Invitrogen), after CDNA synthesis by the MMLV Reverse Transcriptase (Promega). The CFX96 Touch Real-Time PCR Detection System (Bio-Rad) was used, and quantification was performed with the ΔΔCt method. Primer sequences (5′–3′) are provided in Supplemental Table 1.

### L929 cell line and TNF-induced cell death assay.

In the L929 cells (NCTC clone, ATCC), TNF–induced cytotoxicity assay was performed as previously described (80–82).

### In vivo experiments.

All mice were maintained under specific pathogen–free conditions in conventional, temperature-controlled, air-conditioned animal housing facilities of Biomedical Sciences Research Centre “Alexander Fleming” with 12-hour light/12-hour dark cycle. The mice received food and water ad libitum. Similar numbers of female and male mice were used in each experimental group.

### LPS model.

C57BL/6J mice were challenged with 1 μg LPS intraperitoneally. Amisulpride (at 50 mg/kg or 100 mg/kg in water) was administered to C57BL/6J mice by oral gavage 2 hours before (*t* –2) and at the time of LPS administration (*t* 0). Serum samples were collected 1.5 hours after induction in order to measure the levels of IL-6 and murine TNF using the DuoSet (catalog DY406) and eBioscience (catalog 88-7324) ELISAs, respectively, following the manufacturers’ protocols.

### TNF^ΔΑRE^ model.

C57BL/6J *TNF*^ΔΑRE^ (3) mice were administered by oral gavage 20 mg/kg amisulpride diluted in water, twice daily, at 5–12 weeks of age. Arthritis was evaluated clinically in a blinded manner using a semiquantitative arthritis score ranging from 0 to 3, as described elsewhere (6).

### hTNFtg model.

CBA C57BL/6J *hTNFtg* mice (2) were administered by oral gavage 20 mg/kg amisulpride diluted in water, twice daily, either prophylactically at 3–8 weeks of age or therapeutically at 6–10 weeks of age. Arthritis was evaluated clinically in a blinded manner using a semiquantitative arthritis score ranging from 0 to 3, as described elsewhere (6).

### Histology.

Formalin-fixed, EDTA-decalcified, paraffin-embedded mouse joint tissue specimens were sectioned and stained with H&E, toluidine blue (TB), or Tartrate-Resistant Acid Phosphatase (TRAP) Kit (Sigma-Aldrich). H&E and TB were semiquantitatively blindly evaluated for synovial inflammation/hyperplasia (scale of 0–5) and cartilage erosion (scale of 0–5) based on an adjusted, previously described method (83). TRAP staining was performed to measure number of osteoclasts using ImageJ software (NIH) on images acquired with a Nikon microscope, equipped with a QImaging digital camera.

### FACS analysis–based immune infiltration.

FACS analysis–based immune infiltration was assessed by digesting ankle joints of 8-week *hTNFtg* vehicle- or amisulpride-treated animals, with 1,000 U/mL Collagenase IV (Sigma-Aldrich) for 1 hour at 37°C in DMEM, as previously described (27). A total of 1 to 2 million cells were stained according to Supplemental Table 2.

### TNF-TNFRI ELISA.

TNF-TNFRI ELISA was performed as described elsewhere (80).

### Click compound synthesis.

For click 1, the aromatic part of amisulpride (compound h) was synthesized (69), which subsequently reacted with 1-Boc-2-(aminomethyl)pyrrolidine (compound i) under HATU amidation coupling conditions to furnish compound k. Removal of the Boc group followed by reaction with propargyl bromide afforded the desired derivative (Supplemental Figure 7A). Regarding the synthesis of click 2, a slightly different procedure was developed including first the synthesis of the iodide (compound n), which upon palladium-catalyzed multicomponent reductive cross coupling reaction with 4-bromo-1-butyne and sodium metabisulfite as an inorganic sulfur dioxide surrogate (84) afforded compound o. Hydrolysis of the ester derivative followed by amidation coupling with 2-(aminomethyl)-1-ethylpyrrolidine yielded finally the click 2 derivative (Supplemental Figure 7B).

### Mass spectrometry experiments for target identification.

In order to identify the possible targets of amisulpride on *hTNFtg* SFs, a previously described method (85) was employed, by lowering the amount of total protein used to 4 mg. The samples’ digestion, run, and initial analysis were processed as previously described (86). A brief description of the protocol followed in the mass spectrometry experiments is provided in the Supplemental Methods.

### shRNAs and Lenti-X transfection.

For silencing of potential targets of amisulpride, *hTNFtg* SFs were transduced by lentiviruses expressing a full list of shRNAs (described in Supplemental Table 3). shRNAs were subcloned in the HpaI/XhoI sites of pLB vector. Each lentivirus was produced by transient cotransfection with psPAX2 and pMD2G of HEK Lenti-X 293T cell line (Clontech) using polyethylenimine (Sigma, P-3143). Plasmids pLB, psPAX2, and pMD2G were purchased from Addgene. The lentiviral transduction of SFs was performed using Hexadimethrine Bromide/Polybrene (Sigma, H9268), and transduction efficiency was calculated by the FITC-positive cells in a FACSCanto II flow cytometer (BD Biosciences) and FlowJo software (FlowJo, LLC). We sorted the GFP-positive cells, when transfection efficiency was less than 70%. SFs transduced by lentiviruses that carry a vector containing scramble shRNA were used as relevant controls (Supplemental Table 3).

### Phosphoproteomics sample preparation and analysis.

WT SFs activated by hTNF for 5, 15, and 30 minutes with or without 1 hour pretreatment with 500 μM amisulpride in triplicates were processed as previously described (87). Further process details and subsequent analysis of the samples regarding the phosphoproteomics approach are described in Supplemental Methods.

### Adhesion assay.

SFs at 2 × 10^4^ cells/well were allowed to adhere for 30 minutes to a 96-well plate covered with Human Fibronectin (Merck, FC010) at 1 μg/mL. Unbound cells were removed with sequential washes with PBS containing Ca^2+^ and Mg^2+^. Adhered cells were then stained with crystal violet (C3886, Sigma), then solubilized, and their absorbance was determined at 570 nm (80).

### Data and materials availability.

The mass spectrometry raw data have been deposited to the ProteomeXchange Consortium (88) via the PRIDE (89) partner repository with data set identifiers PXD036283 and PXD035230. The RNA-Seq raw data are uploaded at the NCBI Gene Expression Omnibus (90, 91) under accession numbers GSE211132 and GSE211137. The rest of the data are available in the main text or in the supplemental materials.

### Statistics.

All experiments were performed at least 3 times. Data are presented as mean ± SEM. Student’s *t* test (parametric, unpaired, 2-sided) or 1-way ANOVA, followed by Dunnett’s multiple comparisons test, were used for evaluation of statistical significance using GraphPad Prism 8 software. *P* < 0.05 was considered statistically significant.

### Study approval.

All experiments were approved by the Institutional Committee of Protocol Evaluation in conjunction with the Veterinary Service Management of the Hellenic Republic Prefecture of Attica in Attica, Greece, according to all current European and national legislation.

## Author contributions

DP, ANM, and GK conceived the study. DP, FR, CT, PC, AKP, FC, ECV, LN, ANM, and GK developed methodology. DP, FR, CT, PC, AKP, FC, ECV, LN, ANM, and GK investigated. DP, FR, CT, PC, AKP, FC, ECV, LN, ANM, and GK visualized data. GK acquired funding. NK, MCD, JVO, ANM, and GK supervised. DP, ANM, and GK wrote the original draft. All authors reviewed and edited the manuscript.

## Supplementary Material

Supplemental data

Supplemental data set 1

Supplemental data set 2

Supplemental data set 3

Supplemental data set 4

Supplemental data set 5

Supplemental data set 6

Supplemental data set 7

## Version 1 04/04/2023

In-Press Preview

## Version 2 05/08/2023

Electronic publication
