# Supplementary material for: Repurposing the antipsychotic drug amisulpride for targeting synovial fibroblast activation in arthritis
Source: JCI Insight. 2023 May 8;8(9):e165024. doi: 10.1172/jci.insight.165024 (PMC10243819; doi:10.1172/jci.insight.165024)
Supplement: Supplemental data [file jciinsight-8-165024-s079.pdf]

Supplementary Materials and methods

Mass spectrometry experiments for target identification - SP3 digestion and clean up-LC-MS/MS- Data analysis

In brief, after cell treatment and cell lysis, the lysate was subjected to the copper(I)-catalysed alkyne-azide cycloaddition click chemistry approach, for conjugating target proteins with a biotin tag. The biotin-tagged proteins were then pulled down on streptavidin beads, and the target proteins were selectively eluted through cleaving an azo-linker in the tag with sodium dithionite. Finally, the proteins enriched in the eluent were identified by mass spectrometry analysis.

In summary, the eluates were processed using the sensitive sp3 protocol(1), peptide mixtures were collected and were loaded on the trap column at 10uL/min for 4 min with 0.1% formic acid in water and separated in a gradient of 0.1% (vol/vol) formic acid in mobile phase A. and B. acetonitrile.

The data acquisition was performed in positive mode using a Q Exactive HF-X Orbitrap mass spectrometer (ThermoFisher Scientific). MS data were acquired in a data-dependent strategy and the resolution of the survey scan was 120,000 (at m/z 200) with a target value of 3 × 10E6 ions and a maximum injection time of 100 ms.

The acquired raw files were processed through the MaxQuant software (1.6.14.0) using the Mus musculus proteome FASTA database. Perseus (version 1.6.10.43) was used and proteins identified as ‘contaminants’, ‘reverse’ and ‘only identified by site’ were filtered out. The three replicates of each condition were grouped (treated versus vehicle/ competition condition). A two-sided Student’s t-test of the grouped proteins was performed using p-value <0.05 as a significance measurement.

Phosphoproteomics samples processing and analysis

Cell pellets were lysed in buffer containing 5% sodium dodecyl sulfate (SDS), 5 mM tris(2-carboxyethyl)phosphine (TCEP), 10 mM chloroacetamide (CAA), 100 mM Tris, pH 8.5 and boiled for 10’ followed by sonication with a micro tip probe. Protein concentrations were estimated using the Pierce BCA Protein Assay Kit (ThermoFisher Scientific). 500 µg of protein/ sample was used for PAC digestion in an automated 96-well format on a KingFisher™ Flex robot (Thermo Fisher Scientific) with 12 hour O/N digestion at 37 °C, using LysC (Wako) and Trypsin (Sigma Aldrich) as described before(2). Protease activity was quenched by acidification with trifluoroacetic acid (TFA). Peptide mixtures were purified and concentrated on reversed-phase C18 Sep-Pak cartridges (Waters).

For phosphoproteome analysis enrichment of phosphopeptides was carried out in 96-well format on a KingFisher™ Flex robot (Thermo Fisher Scientific) based on previously described protocols(2, 3). Peptides were eluted with 75 µl of 80% acetonitrile directly into a KingFisher 96-well plate and subsequently with 150 µl of loading buffer (80% ACN, 8% TFA and 1.6 M glycolic acid). Phosphopeptides were enriched using TiIMAC-HP beads (MagResyn, Resyn Biosciences) and eluted in the final plate with 1% ammonia. After the eluted phosphopeptides were acidified with TFA, they were directly loaded onto EvoTips according to the manufacturer’s protocol.

All samples were analyzed on the EvoSep One system (using the pre-programmed 60 samples/ day gradient) coupled to an Orbitrap Exploris 480 MS (Thermo Fisher Scientific) through a nanoelectrospray source. Peptides were separated on a 15-cm, 150 µM inner diameter analytical column in-house packed with 1.9 µM reversed-phase C18 beads (ReProsil-Pur AQ, Dr Maisch) and column temperature was maintained at 60°C by an integrated column oven (PRSO-V1, Sonation GmbH). Phosphoproteome analysis was performed using data-independent acquisition (DIA).

All DIA raw files were analyzed using Spectronaut with a library-free approach (directDIA). All files were searched against the mouse UniProt database, supplemented with commonly observed contaminants. For phosphoproteome analysis phosphorylation of serine, threonine

and tyrosine were included as variable modifications and PTM localization cutoff was set to 0.75. Phospho-peptide data was collapsed to site information using the Perseus plugin previously described(4).

DIA phosphoproteome data were processed using R (version 3.6.2) with the Prostar data analysis pipeline(5). Data were log2 transformed and filtered (a minimum of two valid values in at least one condition were required for an identification to be included in downstream analysis). Data were normalized by quantile-based normalization and imputation to replace missing values was performed using a two-step approach(3). Further data analysis of proteomics data was performed using Perseus software version 1.6.2.2 or 1.6.5.0. Data were normalized by row-based median subtraction and heatmaps were generated based on unsupervised hierarchical clustering. Specifically, to assess overall Amisulpride effect, phosphoproteome data were median normalized within groups defining initial treatment (WT, TNF treated) and significantly regulated phosphosites comparing inhibitor (treated and untreated) were identified by t-test using a significance cut-off of 0.05. Volcano plots were generated for visualization of significantly regulated sites identified by Student's t-test (significance cut-off 0.05). Gene ontology (GO) term enrichment analysis and Kyoto Encyclopedia of Genes and Genomes (KEGG) pathway enrichment analysis were performed using DAVID(6, 7) and InnateDb(8), respectively.

Supplementary Materials and methods- Tables

1. Supplementary Table 1- Real time Primers’ sequences

The table below describes the 5’ to 3’ sequences of RT primers used. B2m or Gapdh were used as reference genes.

Gene	5’→ 3’sequence
hTNF	5’-CTTCTCGAACCCCGAGTGAC-3’
Mmp3	5’-GTCTCCCTGCAACCGTGAA-3’
Cxcl3	5’-CTGCACCCAGACAGAAGTCATA-3’
Cox2	5'-TCAGTTTTTCAAGACAGATC-3'
DRD2	5’-GTTTCCCAGTGAACAGGCGG-3’
DRD3	5’-TGGCAACGGTCTGGTATGTG -3’
Htr7	5’-TGCAACGTCTTCATCGCCA-3’
Ascc3	5’-GGCCTTACATGGAAGAAGATAGTG-3’
Kif5c	5’-AGCGGGAAGCTGTATTTGGT-3’
Romo1	5’-GAGCACTCTCGCCGCAGAT-3’
Sec62	5’-GCAGTAATAGCAGCCACCCT-3’
Cdc42	5’-GGCGGAGAAGCTGAGGAC-3’
B2m	5'-TTCTGGTGCTTGTCTCACTGA-3'
Gapdh	5’-GTTGTCTCCTGCGACTTCA-3’

Supplementary Table 1: 5’ to 3’ sequences of RT primers used in the study.

2. Supplementary Table 2- Antibodies used in Immune infiltration FACs analysis

Unspecific binding was blocked by the anti-Fc Receptor (anti-CD16/32) antibody (Biolegend). Analysis was performed using a FACS Canto II Flow cytometer (BD Biosciences) and FlowJo software (FlowJo, LLC). Counting beads were used for quantification of different cell subsets. Supplementary Table 2 summarizes the antibodies used for FACs staining. FACs gating was performed as previously described(9).

**Cells subset**	**Antibodies (Company, Cat. Number)**								**Live/ Dead Exclusion**
**Myeloid cells**	PE-conjugated anti-CD11b (BD Biosciences, 557397)	A700-conjugated anti-CD45 (Biolegend, 103128)	APC-conjugated anti-MHCII (eBioscience,17-5320-82)	PE/Dazzle594-conjugated anti-CD64 (Biolegend, 139320)	APCFire-conjugated anti-CD24 (Biolegend,101840)	PE/Cy7-conjugated anti-CD11c (Biolegend, 117318)	FITC-conjugated anti-Ly6C (BD Biosciences, 553104)	Biotinylated anti-Ly6G (eBioscience, 13-5931-75) with streptavidin-conjugated PE/Cy5 (Invitrogen)	Dapi (Invitrogen, D1306)
**Lymphocytes**	PE-conjugated anti-B220 (BD Biosciences, 553089)	ApcCy7-conjugated anti-CD45 (Biolegend, 103116)	PE/Cy7-conjugated anti-CD3 (eBioscience,25-0031-82)	A700-conjugated anti-CD4 (Biolegend, 100536)	APC-conjugated anti-CD8 (Biolegend,100711)				Zombie Green (Sigma, 423112)
**Fibroblasts**	A488-conjugated anti-CD90.2 (Biolegend, 105316)	A700-conjugated anti-CD45 (Biolegend, 103128)	PE-conjugated anti-CD31 (BD Biosciences, 553373)	PE/Cy7-conjugated anti-PDPN (Biolegend, 127412)					Zombie NIR (Sigma, 77184)

Supplementary Table 2: Antibodies used in Immune infiltration FACs analysis

3. Supplementary Table 3- ShRNAs sequences for Lentiviral vectors creation

Gene to silence	shRNA sequence
Ascc3	5’ T-GGAAGAAGATAGTGAAATT-TTCAAGAGA-AATTTCACTATCTTCTTCC-TTTTTTC 3’
	3’ A-CCTTCTTCTATCACTTTAA-AAGTTCTCT-TTAAAGTGATAGAAGAAGG-AAAAAAGAGCT 5’
Kif5c	5’ T-GCAAAGACCATCAAGAATA-TTCAAGAGA-TATTCTTGATGGTCTTTGC-TTTTTTC 3’
	3’ A-CGTTTCTGGTAGTTCTTAT-AAGTTCTCT-ATAAGAACTACCAGAAACG-AAAAAAGAGCT 5’
Romo1	5’ T-GCAAAGACCATCAAGAATA-TTCAAGAGA-TATTCTTGATGGTCTTTGC-TTTTTTC 3’
	3’A-CCCTTTTGGTACTACGTCT-AAGTTCTCT-AGACGTAGTACCAAAAGGG-AAAAAAGAGCT 5’
Sec62	5’ T-GGGATTAATTCTTGTGATT-TTCAAGAGA-AATCACAAGAATTAATCCCT-TTTTTC 3'
	3’ A-CCCTAATTAAGAACACTAA-AAGTTCTCT-TTAGTGTTCTTAATTAGGG-AAAAAAGAGCT 5’
Cdc42	5’ T-GCAAGAGGATTATGACAGA-TTCAAGAGA-TCTGTCATAATCCTCTTGC-TTTTTTC 3’
	3’ A-CGTTCTCCTAATACTGTCT-AAGTTCTCT- AGACAGTATTAGGAGAACG-AAAAAAGAGCT 5’
Scramble	5' T-GCAGTGCAATATCGGAAAC-TTCAAGAGA-GTTTCCGATATTGCACTGCTTTTTTC 3’
	3’ A-CGTCACGTTATAGCCTTTG-AAGTTCTCT-CAAAGGCTATAACGTGACG-AAAAAAGAGC 5’

Supplementary Table 3: shRNAs sequences used in the study.

Supplementary Figures

Fig. S1

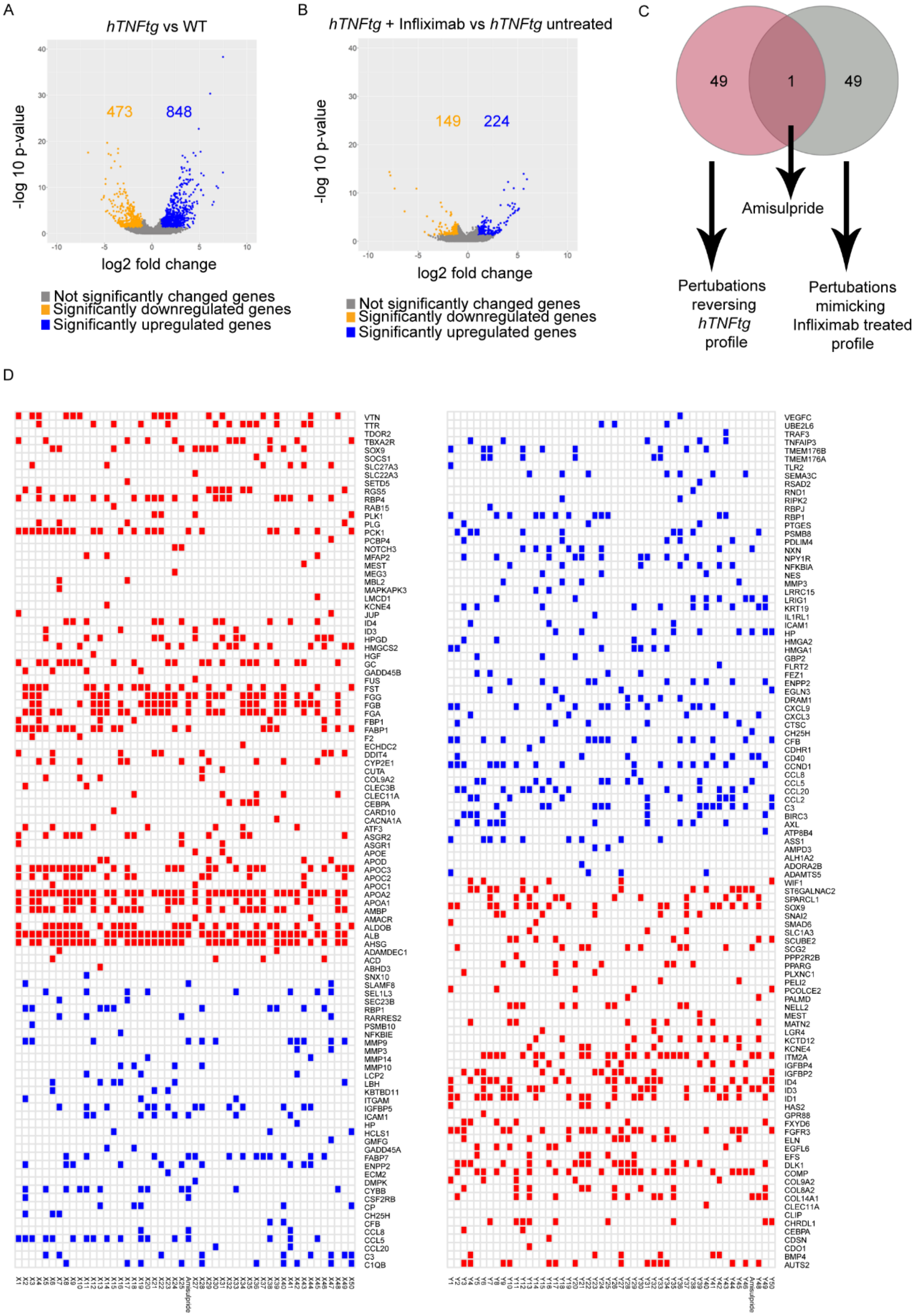

Fig. S1. **Methodology for the identification of new candidates for the deactivation of *hTNFtg* fibroblasts.** **(A)** Volcano plot showing the number of deregulated genes in *hTNFtg* SFs versus WT control (n=3) **(B)** Volcano plot showing the number of deregulated genes in *hTNFtg* SFs before and after 48h treatment with Infliximab at 1μg/ml (n=3) **(C)** Venn diagram showing the overlap between perturbations that reverse the *hTNFtg* profile and mimic the Infliximab-treated disease signature **(D)** Heatmaps presenting the top 50 perturbations proposed by the L1000CDS^2 search engine to reverse the *hTNFtg* profile or to mimic the Infliximab-treated disease signature, respectively (see also Data file 3)

Fig. S2

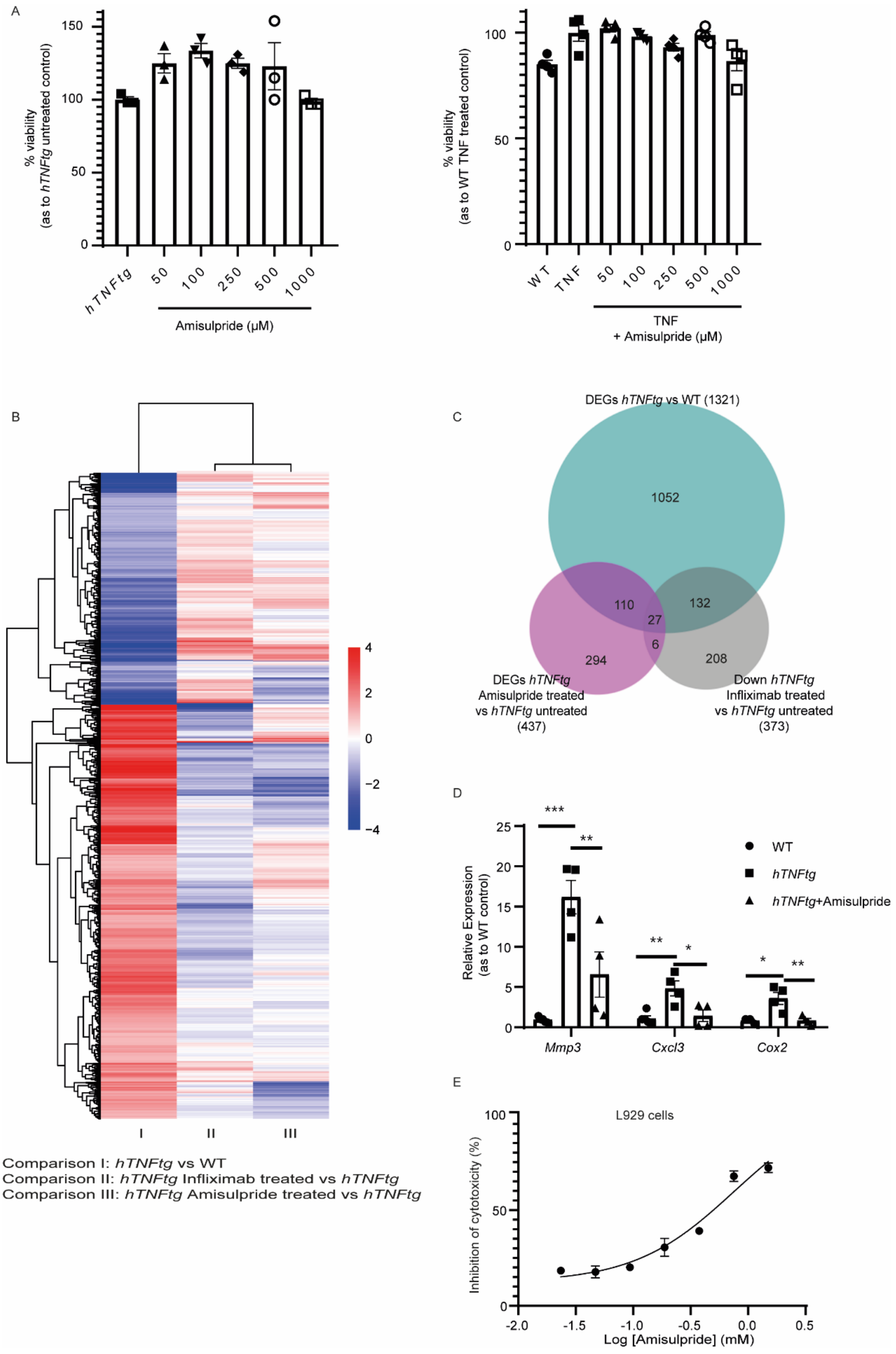

Fig. S2. **Ex vivo effect of Amisulpride (A)** Crystal violet viability assay of *hTNFtg*, WT and WT TNF treated SFs upon Amisulpride in the indicated concentrations for 48h, compared with the vehicle treated relevant controls (n=3-4). **(B)** Heatmap of the 1321 genes identified to be deregulated in *hTNFtg* vs

WT SFs. Amisulpride affects 437 and Infliximab 373 of these genes (n=3) **(C)** Venn diagram presenting the total number of genes being deregulated in *hTNFtg* vs WT SFs while being affected upon Amisulpride/ Infiliximab treatment (n=3) **(D)** qPCR analysis of *Mmp3*, *Cxcl3* and *Cox2* in WT SFs, *hTNFtg* SFs and *hTNFtg* SFs treated with 500μM Amisulpride for 48h (n=4) **(E)** Crystal violet based quantification of L929 cells TNF-induced necroptosis inhibition upon ex vivo Amisulpride treatment in a dose-dependent manner (n=3) (* p-value < 0.05; ** p-value < 0.01; *** p-value ≤ 0.0001, all data are shown as mean ± SEM, statistics are performed using One- way Anova, followed by Dunnett's multiple comparisons test)

Fig. S3

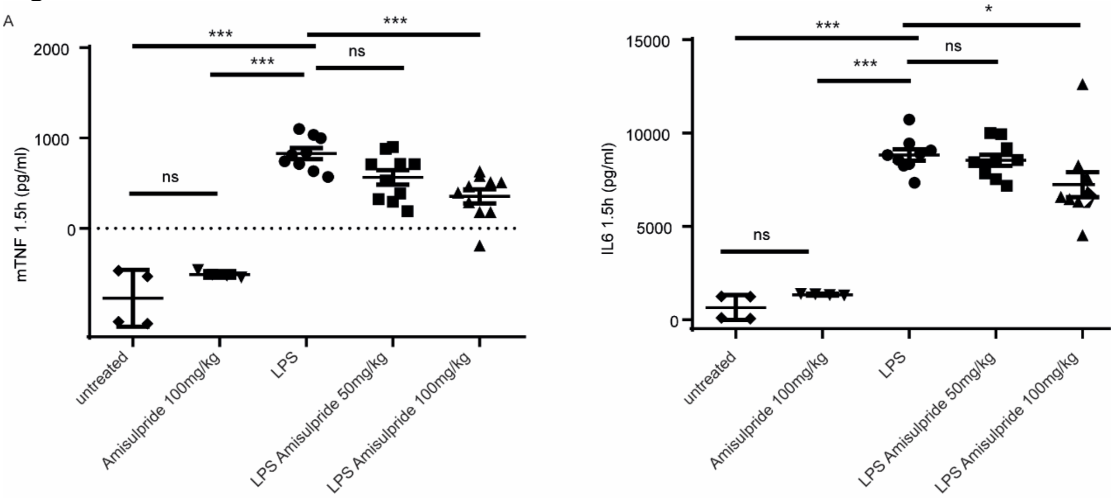

Fig. S3. Amisulpride alleviates endotoxemia in vivo (A) mTNF and IL6 quantification in the serum of Amisulpride treated mice 1,5h after sepsis induction using 1μg LPS intraperitoneally (Amisulpride administered in the indicated concentrations, 2 hours before and at the time of LPS administration) (n=4-10) (* p-value < 0.05; ** p-value < 0.01; *** p-value ≤ 0.0001, all data are shown as mean ± SEM, statistics are performed using Student's t test or One- way Anova, followed by Dunnett's multiple comparisons test)

Fig. S4

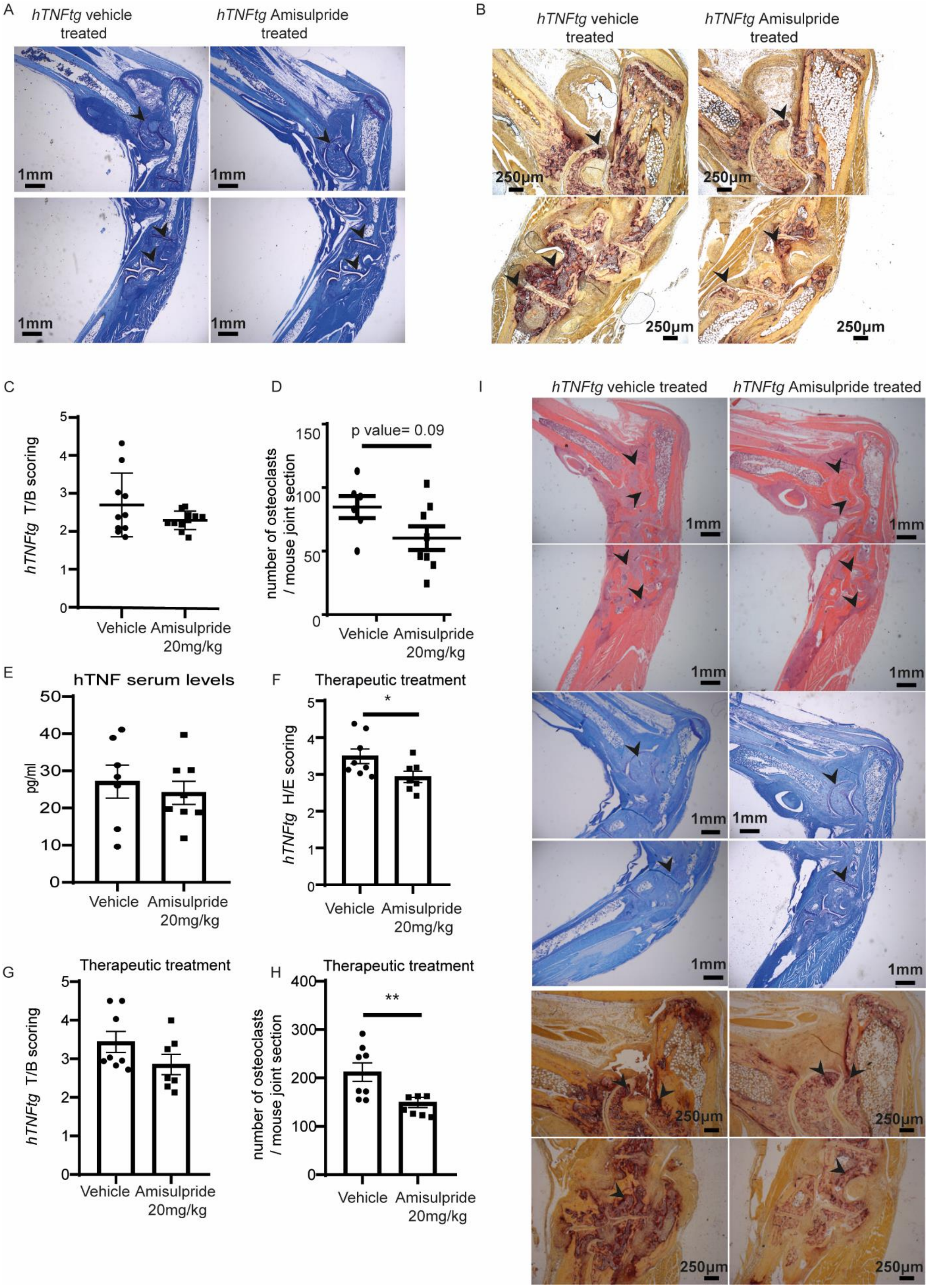
A
hTNFtg vehicle treated
hTNFtg Amisulpride treated
1mm
B
hTNFtg vehicle treated
hTNFtg Amisulpride treated
250µm
C
hTNFtg T/B scoring
Vehicle
Amisulpride 20mg/kg
D
p value= 0.09
number of osteoclasts / mouse joint section
Vehicle
Amisulpride 20mg/kg
I
hTNFtg vehicle treated
hTNFtg Amisulpride treated
1mm
250µm
E
hTNF serum levels
pg/ml
Vehicle
Amisulpride 20mg/kg
F
Therapeutic treatment
*
hTNFtg H/E scoring
Vehicle
Amisulpride 20mg/kg
G
Therapeutic treatment
hTNFtg T/B scoring
Vehicle
Amisulpride 20mg/kg
H
Therapeutic treatment
**
number of osteoclasts / mouse joint section
Vehicle
Amisulpride 20mg/kg

Fig. S4. In vivo effect of Amisulpride on the *hTNFtg* polyarthritis model Representative histological images of **(A)** T/B (original magnification 2X, scale bar= 1mm) and **(B)** TRAP stained paraffin sections (original magnification 4X, scale bar= 250μm) of ankle joints of *hTNFtg* mice treated prophylactically with 20mg/kg Amisulpride by oral gavage (week 3- week 8) when compared with the vehicle-treated controls. The sections in the top and bottom row show ankle and metatarsal field of the same representative section and black arrows indicate examples of regions of interest **(C)** T/B scoring of paraffin sections of ankle joints of *hTNFtg* treated prophylactically with 20mg/kg Amisulpride (week 3- week 8) when compared with the vehicle-treated controls (n=10) **(D)** Osteoclasts' numbers counted out of TRAP stained paraffin sections of ankle joints of *hTNFtg* treated prophylactically with 20mg/kg Amisulpride (week 3- week 8) when compared with the vehicle-treated controls (n=6-8) **(E)** hTNF serum levels of *hTNFtg* mice treated with 20mg/kg Amisulpride for 5weeks (week 3- week 8) (n=6-8) **(F)** H/E scoring, **(G)** T/B scoring and **(H)** osteoclasts' numbers counted on TRAP stained slides of paraffin sections of ankle joints of *hTNFtg* treated therapeutically with 20mg/kg Amisulpride by oral gavage (week 6- week 10) when compared with the vehicle-treated controls (n=7-8) **(I)** Representative histological images of H/E and T/B (original magnification 2X, scale bar= 1mm) and TRAP stained paraffin sections (original magnification 4X, scale bar= 250μm) of ankle joints of *hTNFtg* mice treated therapeutically with 20mg/kg Amisulpride by oral gavage (week 5- week 10) when compared with the vehicle-treated controls (n=7-8). The sections in the top and bottom row show ankle and metatarsal field of the same representative section and black arrows indicate examples of regions of interest (* p-value < 0.05; ** p-value < 0.01; *** p-value ≤ 0.0001, all data are shown as mean $\pm$ SEM, statistics are performed using Student's t test)

Fig. S5

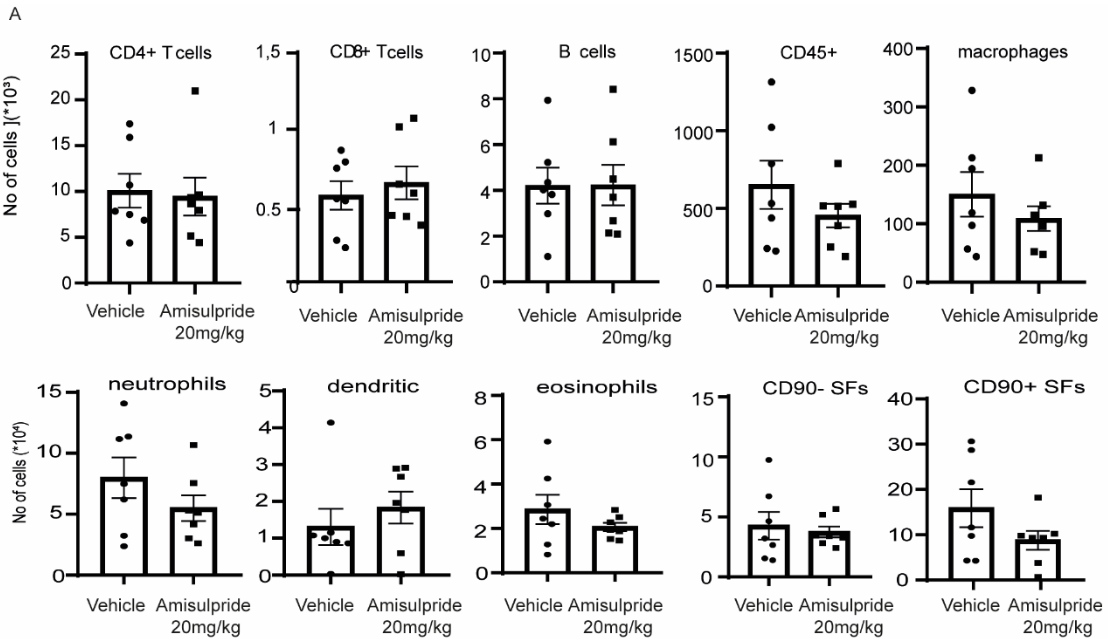

Fig. S5. Quantification of cells number in *hTNFtg* Amisulpride- treated mice (A) Immune infiltration and fibroblasts numbers FACs analysis of ankle joints of *hTNFtg* mice treated prophylactically with 20mg/kg Amisulpride by oral gavage for 5weeks (week 3- week 8), compared with the vehicle treated control (n=6-7) (* p-value < 0.05; ** p-value < 0.01; *** p-value ≤ 0.0001, all data are shown as mean ± SEM, statistics are performed using Student’s t test)

120

Fig. S6

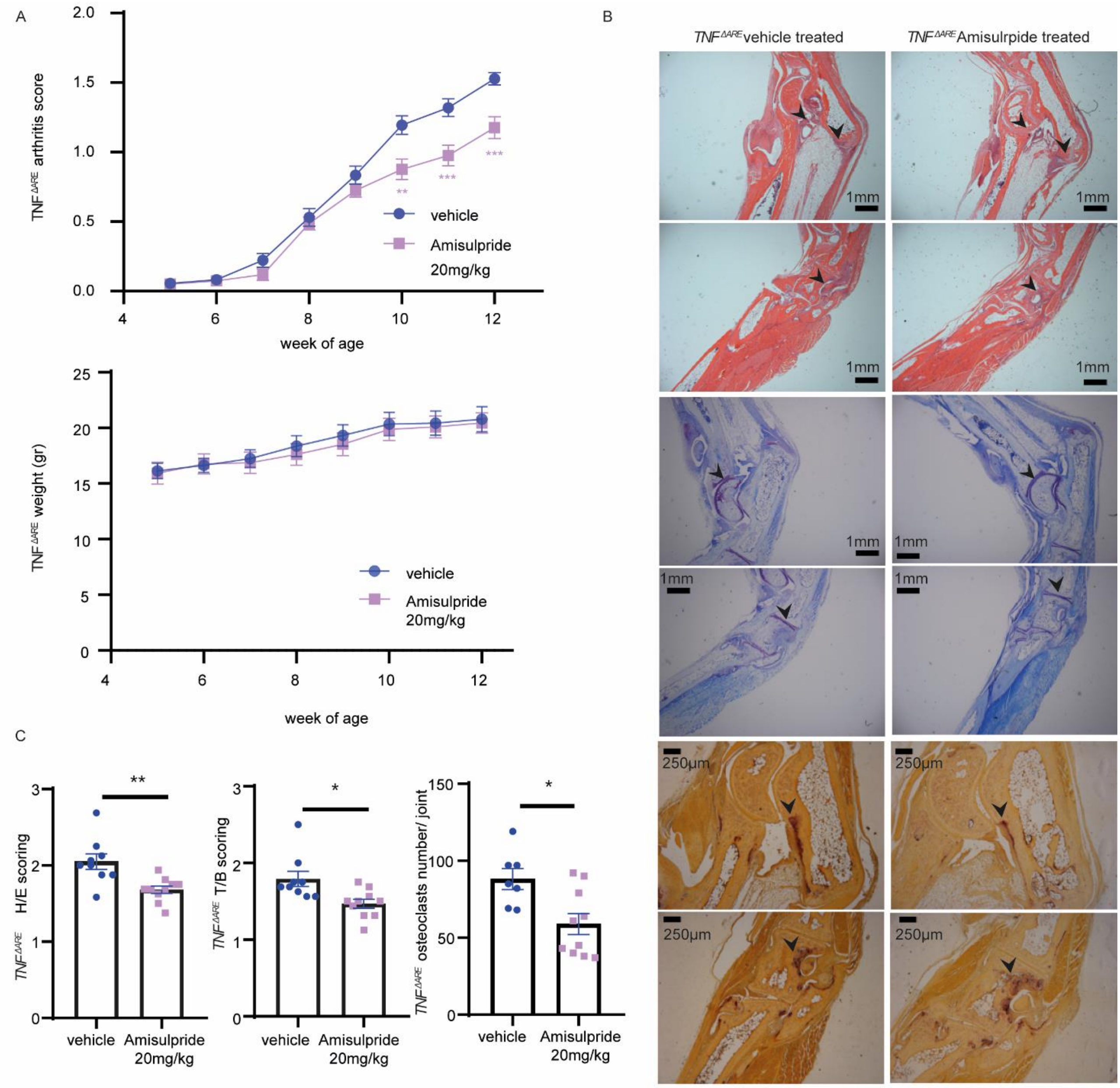

Fig. S6 In vivo effect of Amisulpride on the *TNF$^{\Delta ARE}$* polyarthritis model (A) Arthritis clinical score and weight measurement of *TNF$^{\Delta ARE}$* mice treated by oral gavage with 20mg/kg Amisulpride (week 5- week 12) when compared with the vehicle-treated controls (n=10) **(B)** Representative histological images of H/E, T/B (original magnification 2X, scale bar = 1mm) and Trap stained (original magnification 4X, scale bar = 250μM) paraffin sections of joints of *TNF$^{\Delta ARE}$* mice treated with 20mg/kg Amisulpride by oral gavage (week 5- week 12) when compared with the vehicle-treated controls (n=7-10). The sections in the top and bottom row show ankle and metatarsal field of the same representative section and black arrows indicate examples of regions of interest **(C)** Synovitis, T/B scoring and osteoclasts number in histological images of H/E, T/B and Trap stained paraffin sections of joints of *TNF$^{\Delta ARE}$* mice treated with 20mg/kg Amisulpride by oral gavage (week 5- week 12) when compared with the vehicle-treated controls (n=7-10) (* $p\text{-value} < 0.05$; ** $p\text{-value} < 0.01$; *** $p\text{-value} \leq 0.0001$, all data are shown as mean ± SEM, statistics are performed using Student's t test)

Fig. S7

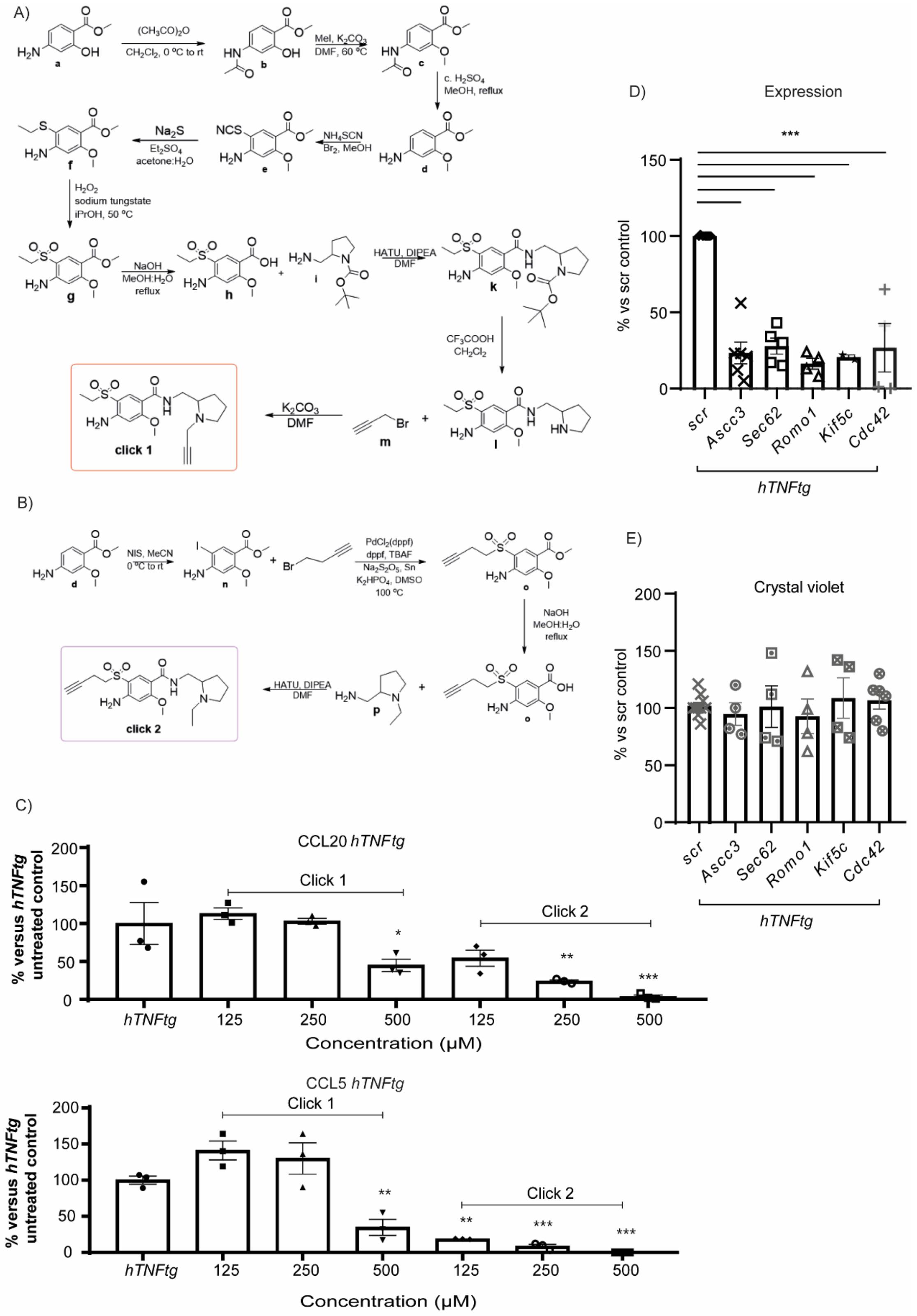

Fig. S7. **Amisulpride targets' identification on *hTNFtg* SFs** Synthetic routes followed for the synthesis of compounds **(A)** click 1 and **(B)** click 2. **(C)** Expression of CCL20 and CCL5 upon different concentrations of the click compound 1 and click compound 2 on *hTNFtg* arthritic SFs when compared with the vehicle treated control (n=3) **(D)** Expression of *Ascc3*, *Sec62*, *Romo1*, *Kif5c* and *Cdc42* upon Lentiviral transfection of *hTNFtg* SFs with the respective shRNAs targeting Amisulpride potential targets when compared with the scramble treated control (n=3-6) **(E)** Crystal violet assay upon shRNAs

transfection targeting Amisulpride potential targets when compared with the scramble treated control (n=3-6) (* $p\text{-value} < 0.05$; ** $p\text{-value} < 0.01$; *** $p\text{-value} \leq 0.0001$, all data are shown as mean ± SEM, statistics are performed using One -way Anova, followed by Dunnett's multiple comparisons test)

Fig. S8

A

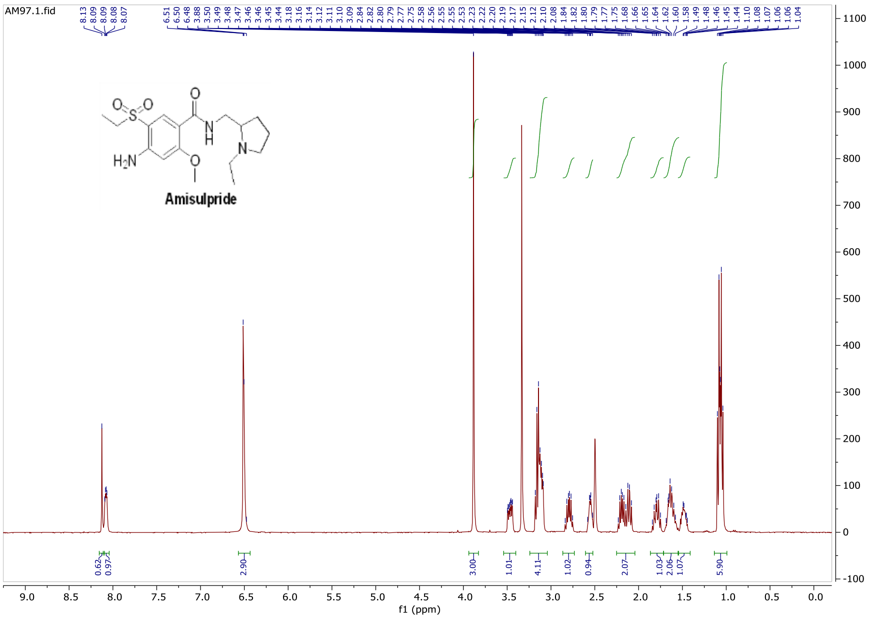

^{1}H-NMR (400 MHz, dmso-d$_6$) δ 1.07 (dt, J_1 = 10.6 Hz, J_2 = 7.2 Hz, 6H), 1.44-1.49 (m, 1H), 1.58-1.68 (m, 2H), 1.75-1.84 (m, 1H), 2.08-2.23 (m, 2H), 2.53-2.58 (m, 1H), 2.75-2.84 (m, 1H), 3.09-3.18 (m, 4H), 3.47 (ddd, J_1 = 13.4 Hz, J_2 = 6.9 Hz, J_3 = 2.9 Hz, 1H), 3.88 (s, 3H), 6.48-6.51 (m, 3H), 8.07-8.09 (m, 1H), 8.13 (s, 1H). MS [ESI$^+$] m/z: 370.5 [M + H$^+$]$^+$.

B

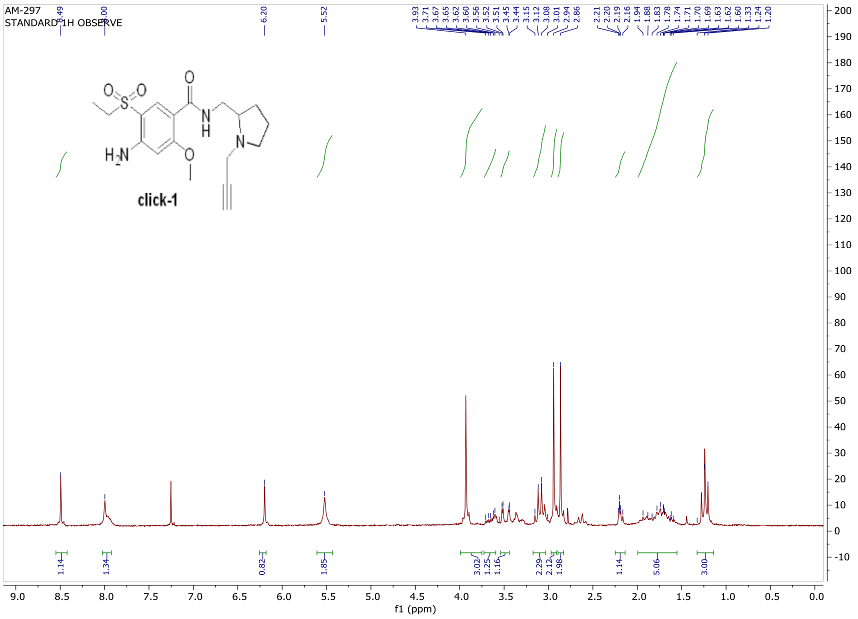

^{1}H-NMR (200 MHz, CDCl$_3$) δ 1.24 (t, J = 7.2 Hz, 3H), 1.60-1.94 (m, 5H), 2.16-2.21 (m, 1H), 2.86 (s, 2H), 2.94 (s, 2H), 3.01-3.15 (m, 2H), 3.44-3.52 (m, 1H), 3.56-3.71 (m, 1H), 3.93 (s, 3H), 5.52 (brs, 2H), 6.20 (s, 1H), 8.00 (brs, 1H), 8.49 (s, 1H). MS [ESI$^+$] m/z: 380.6 [M + H$^+$]$^+$.

C

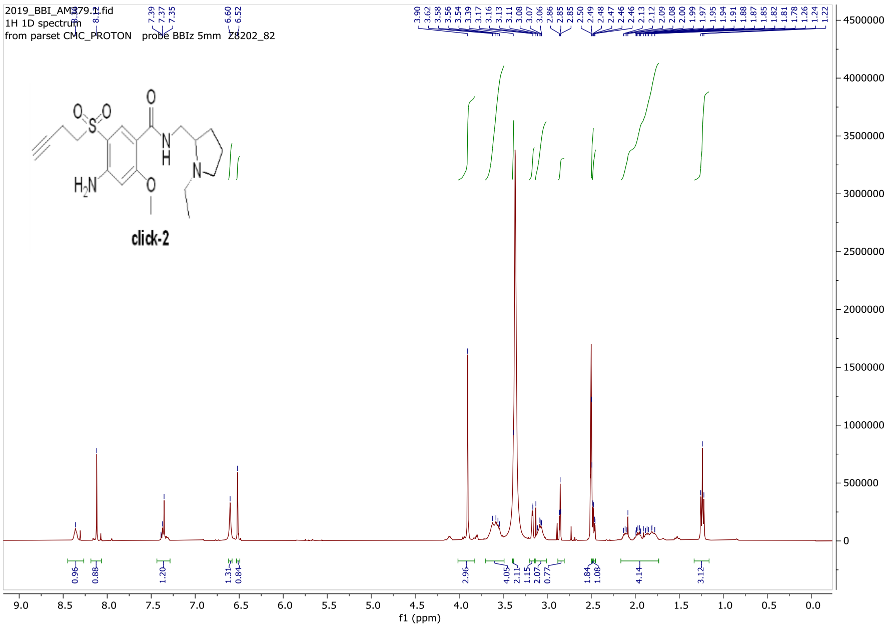

^{1}H-NMR (400 MHz, dmso-d$_6$) δ 1.24 (t, J = 7.2 Hz, 3H), 1.78-2.13 (m, 4H), 2.47 (dd, J_1 = 7.4 Hz, J_2 = 2.8 Hz, 1H), 2.48-2.50 (m, 2H), 2.85 (t, J = 2.7 Hz, 1H), 3.06-3.13 (m, 2H), 3.17 (d, J = 3.6 Hz, 1H), 3.39 (m, 2H), 3.54-3.62 (m, 4 H), 3.90 (s, 3H), 6.52 (s, 1H), 6.60 (s, 1H), 7.35-7.39 (m, 1H), 8.12 (s, 1H), 8.30 (s, 1H). MS [ESI$^+$] m/z: 394.18 [M + H$^+$]$^+$.

Fig. S8. ^{1}H-NMR spectra for the synthesized **(A)** Amisulpride, **(B)** click-1 and **(C)** click-2

Fig. S9

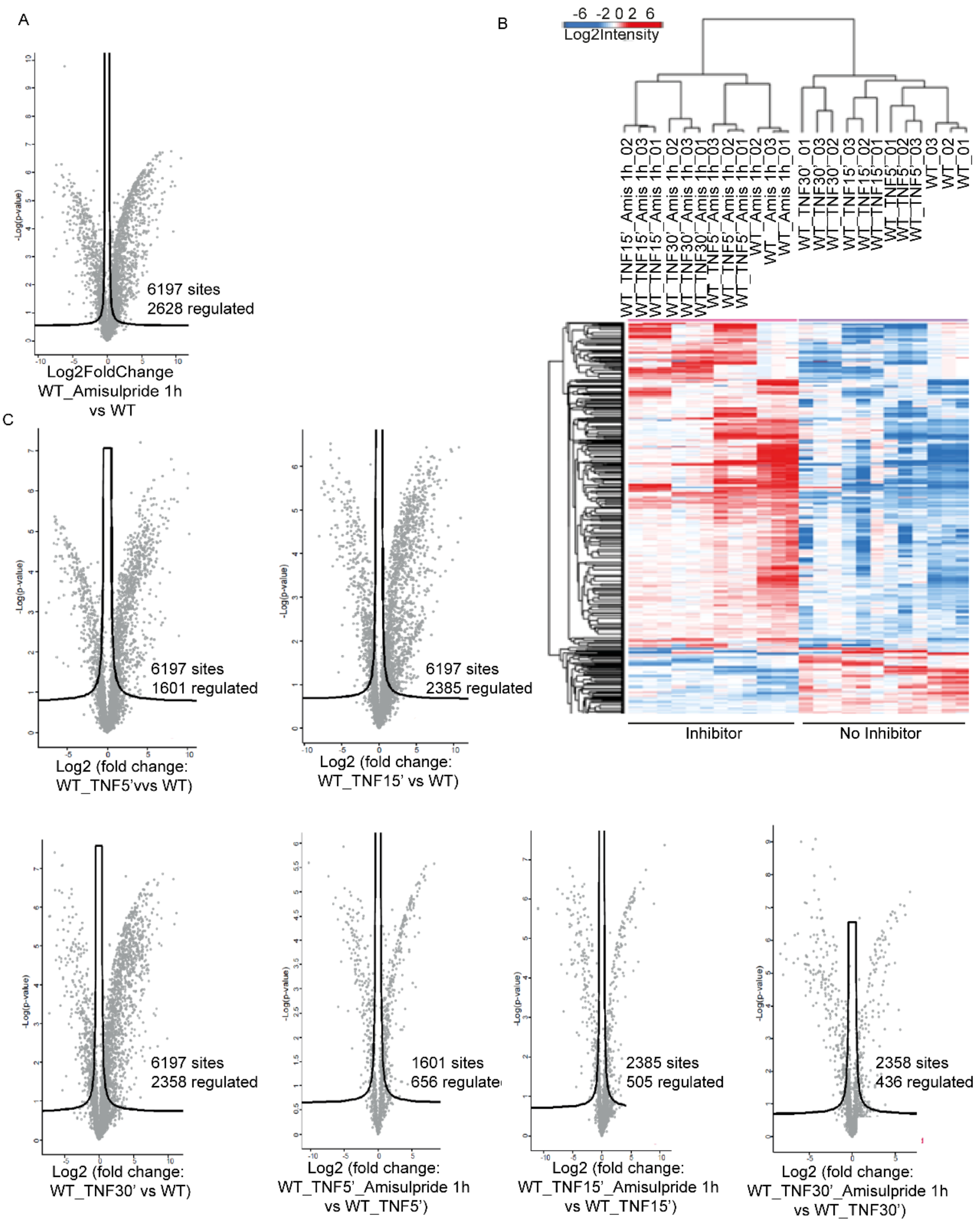

Fig. S9. **Phosphoproteomics analysis of intraarticular SFs treated with Amisulpride (A)** Volcano plot illustrating phosphorylations that are up and downregulated upon Amisulpride treatment with significance cut-off = 0.05 indicated by lines (n=3) **(B)** Heatmap diagram based on hierarchical clustering analysis of t-test significantly regulated phosphosites comparing Amisulpride treated and untreated SFs (n=3) **(C)** Volcano plots with significance cut-off = 0.05 indicated by lines, showing the deregulation of phosphoproteome on hTNF stimulated WT SFs (5', 15' and 30') when compared with untreated controls and the deregulation of phosphosites at samples pre-treated with Amisulpride at 500μM, 1 hour before TNF stimulation when compared with the relevant TNF treated control (n=3)

Supplementary Data Files

Data File 1-filtered_all_out_WU_vs_TU- RNA sequencing data hTNFtg SFs (TU) vs WT SFs (WU)

Data File 2-filtered_all_out_TU_vs_TR- RNA sequencing data of hTNFtg SFs Infliximab treated (TR) vs vehicle-treated (TU)

Data file 3_LINCS1000_supplementary_table- input files and results

Data File 4-filtered_all_out_T_vs_TAM- RNA sequencing data of hTNFtg SFs Amisulpride (TAM) treated vs vehicle-treated (T)

Data File 5- Click proteomics data

Data File 6- Phosphoregulations for WT samples treated with Amisulpride vs Untreated vehicle controls (used for Heatmap in Figure S9B)

Data File 7-Phosphoregulations of WT samples treated with TNF and Amisulpride vs Phosphoregulations of WT samples treated only with TNF (Figure 6A and 6B)

References- Supplementary Material

1. C. S. Hughes, S. Foehr, D. A. Garfield, E. E. Furlong, L. M. Steinmetz, J. Krijgsveld, Ultrasensitive proteome analysis using paramagnetic bead technology. *Mol. Syst. Biol.* (2014), doi:10.15252/msb.20145625.

2. D. B. Bekker-Jensen, A. Martínez-Val, S. Steigerwald, P. Rüther, K. L. Fort, T. N. Arrey, A. Harder, A. Makarov, J. V. Olsen, A compact quadrupole-orbitrap mass spectrometer with FAIMS interface improves proteome coverage in short LC gradients. *Mol. Cell. Proteomics* (2020), doi:10.1074/mcp.TIR119.001906.

3. A. Martinez-Val, D. B. Bekker-Jensen, S. Steigerwald, C. Koenig, O. Østergaard, A. Mehta, T. Tran, K. Sikorski, E. Torres-Vega, E. Kwasniewicz, S. H. Brynjólfsdóttir, L. B. Frankel, R. Kjøbsted, N. Krogh, A. Lundby, S. Bekker-Jensen, F. Lund-Johansen, J. V. Olsen, Spatial-proteomics reveals phospho-signaling dynamics at subcellular resolution. *Nat. Commun. 2021 121* (2021).

4. D. B. Bekker-Jensen, O. M. Bernhardt, A. Hogrebe, A. Martinez-Val, L. Verbeke, T. Gandhi, C. D. Kelstrup, L. Reiter, J. V. Olsen, Rapid and site-specific deep phosphoproteome profiling by data-independent acquisition without the need for spectral libraries. *Nat. Commun.* (2020), doi:10.1038/s41467-020-14609-1.

5. S. Wieczorek, F. Combes, C. Lazar, Q. G. Gianetto, L. Gatto, A. Dorffer, A. M. Hesse, Y. Couté, M. Ferro, C. Bruley, T. Burger, DAPAR & ProStaR: Software to perform statistical analyses in quantitative discovery proteomics. *Bioinformatics* (2017), doi:10.1093/bioinformatics/btw580.

6. D. W. Huang, B. T. Sherman, R. A. Lempicki, Systematic and integrative analysis of large gene lists using DAVID bioinformatics resources. *Nat. Protoc.* (2009), doi:10.1038/nprot.2008.211.

7. D. W. Huang, B. T. Sherman, R. A. Lempicki, Bioinformatics enrichment tools: Paths toward the comprehensive functional analysis of large gene lists. *Nucleic Acids Res.* (2009), doi:10.1093/nar/gkn923.

8. D. J. Lynn, G. L. Winsor, C. Chan, N. Richard, M. R. Laird, A. Barsky, J. L. Gardy, F. M. Roche, T. H. W. Chan, N. Shah, R. Lo, M. Naseer, J. Que, M. Yau, M. Acab, D. Tulpan, M.

D. Whiteside, A. Chikatamarla, B. Mah, T. Munzner, K. Hokamp, R. E. W. Hancock, F. S. L. Brinkman, InnateDB: Facilitating systems-level analyses of the mammalian innate immune response. *Mol. Syst. Biol.* (2008), doi:10.1038/msb.2008.55.

9. F. Roumelioti, C. Tzaferis, D. Konstantopoulos, D. Papadopoulou, A. Prados, M. Sakkou, A. Liakos, P. Chouvardas, T. Meletakos, Y. Pandis, N. Karagianni, M. Denis, M. Fousteri, M. Armaka, G. Kollias, miR-221 / 222 drive synovial fibroblast expansion and pathogenesis of TNF-mediated arthritis. *bioRxiv* (2022).
